# Overcorrection of severe hyponatremia, osmotic demyelination syndrome, and mortality: insights from two Brazilian centers

**DOI:** 10.1590/2175-8239-JBN-2025-0161en

**Published:** 2026-01-23

**Authors:** Lívia de Azevêdo Cerqueira Reis, Maria Gabriela Motta Guimarães, Ananda Pires Bastos, Dyonatas Rodrigues da Mata, Daniel Henrique Lins e Silva, Paulo Novis Rocha

**Affiliations:** 1 Universidade Federal da Bahia, Programa de Pós-Graduação em Ciências da Saúde, Salvador, BA, Brazil.; 2 Universidade Federal da Bahia, Faculdade de Medicina da Bahia, Salvador, BA, Brazil.; 3 Universidade Federal da Bahia, Faculdade de Medicina da Bahia, Departamento de Medicina Interna e Apoio Diagnóstico, Salvador, BA, Brazil.

**Keywords:** Hyponatremia, Myelinolysis, Central Pontine, Saline Solution, Inappropriate ADH Syndrome

## Abstract

**Introduction:**

Severe hyponatremia (sodium ≤ 120 mmol/L) poses significant clinical risks, including encephalopathy and seizures, but inadvertent rapid correction may cause osmotic demyelination syndrome (ODS). Current guidelines recommend limiting sodium correction to ≤8 mmol/L per 24 hours to minimize ODS risk. However, recent studies suggest that overcorrection may not directly contribute to mortality and could even be associated with improved outcomes.

**Methods:**

This retrospective cohort study included 362 patients with severe hyponatremia admitted to two Brazilian tertiary hospitals. Overcorrection was defined as a serum sodium increase >8 mmol/L in 24 hours or >18 mmol/L in 48 hours. Multivariate logistic regression and propensity score-weighted analyses were used to identify predictors and outcomes associated with overcorrection.

**Results:**

Overcorrection occurred in 38.7% of patients whereas ODS occurred in only one patient (0.28%). Independent predictors of overcorrection included younger age, lower admission sodium levels, and higher volumes of 0.9% NaCl administered in the emergency room; cancer diagnosis and furosemide use were protective factors. Overcorrection was associated with lower in-hospital mortality and shorter hospital stays, even in propensity score-weighted multivariate analyses. However, a detailed review of mortality cases revealed no direct causal link between the rate of sodium correction and death.

**Conclusion:**

Overcorrection of severe hyponatremia was common and associated with better clinical outcomes, without a significant increase in the risk of ODS. However, given the observational nature of this association, randomized controlled trials are needed before the current guidelines for correction rate can be reconsidered.

## Introduction

When water intake exceeds renal excretion, patients develop hyponatremia. Since serum sodium concentration determines the tonicity of the extracellular fluid (ECF), hyponatremia creates a hypotonic state that leads to water movement from the ECF to the intracellular fluid (ICF). Severe hyponatremia, defined by a serum sodium concentration < 120 mmol/L, is particularly dangerous and can result in encephalopathy, coma, seizures, and death. As a protective mechanism, neurons adapt by reducing their electrolyte and osmolyte content to minimize water influx, thereby stabilizing cellular volume. However, these adaptations take time both to develop and to reverse. When chronic hyponatremia is corrected too quickly, the relatively hypertonic ECF can draw water out of neurons, causing a rapid reduction in neuron volume. In susceptible patients, this can lead to osmotic demyelination syndrome (ODS)^1^.

The first case report of ODS was published in 1959 by Adams et al.^2^, and since then, animal models have confirmed the role of rapid correction of hyponatremia and its pathogenesis^3,4^. A recent systematic review demonstrated that the median serum sodium in 96 confirmed ODS cases at admission was 105 mmol/L, with over 90% of cases having serum sodium levels < 120 mmol/L with correction rates > 10 mmol/L/day^5^. Given the risks posed by both severe hyponatremia itself and treatment-related ODS, the management of hyponatremia has been a long-standing subject of debate^6,7^.

In the past, controversy over the rate of correction of severe hyponatremia led to significant disagreements among experts. Some emphasized the risks of hyponatremic encephalopathy and advocated for faster correction^8,9,10^, while others expressed more concern about ODS and recommended caution in correcting severe hyponatremia^11,12,13^. Over time, a more conservative approach has prevailed. The recommended rate of correction has gradually decreased from 1 mmol/L/hour to < 0.5 mmol/L/hour. The 2013 American guidelines proposed a target of 6 mmol/L/day for patients at significant risk for ODS, with a limit of 8 mmol/L/day^14^.

Limiting the correction of severe hyponatremia to < 8 mmol/L/day can be challenging, as serum sodium levels can rise unintentionally in patients with reversible causes of hyponatremia. For example, in hypovolemic hyponatremia, volume replacement suppresses baroreceptor-mediated antidiuretic hormone (ADH) secretion, triggering water diuresis and an unintentional, rapid rise in serum sodium^15^. A large multicenter American study indicated that overcorrection occurs in approximately 40% of patients, with risk factors including younger age, female gender, schizophrenia, severe hyponatremia, and initial low urinary sodium^16^. Overcorrection of severe hyponatremia is considered a medical emergency^17^ and management strategies, such as discontinuing sodium chloride administration and rediluting serum sodium with 5% dextrose in water (D5W)^14^ and, if necessary, giving desmopressin (DDAVP), are outlined in recent guidelines. Some experts have even suggested the prophylactic use of DDAVP in selected cases to prevent aquaresis and overcorrection^18^.

Latin America studies on the frequency of hyponatremia overcorrection are lacking, and differences in practice patterns and in familiarity with hyponatremia guidelines may lead to variability in the incidence of overcorrection across regions. Therefore, the initial objective of our study was to evaluate the incidence and risk factors for overcorrection of severe hyponatremia in two tertiary hospitals in Brazil. However, recent publications in the *NEJM Evidence* have indicated that, while overcorrection of hyponatremia is common, ODS is extremely rare^19,20^. Moreover, patients with severe hyponatremia who experienced serum sodium correction > 10 mmol/L/day—rates considered overcorrection by current standards—had lower in-hospital and 30-day mortality than those corrected more slowly, in line with current guidelines^20^. These findings have reignited a debate, with some authors suggesting that current guidelines may be overly conservative, potentially placing patients at higher risk of mortality due to undercorrection^21,22^, while others maintain that more evidence from randomized trials is needed before changing the guidelines^23^.

Considering these recent findings, our study also aimed to contribute to the ongoing debate by comparing adjusted in-hospital mortality between patients who experienced overcorrection and those who followed the guidelines. Additionally, we sought to determine the immediate cause of death to assess whether hyponatremia or its rate of correction played a causal role.

## Methods

Study Design and Sample Selection: This was a retrospective cohort study that analyzed the electronic medical records of patients from two tertiary healthcare institutions, one philanthropic and the other private, both located in Salvador, Bahia, Brazil. Initially, we requested a list of all patients who had severe hyponatremia, defined as an initial serum sodium value of < 120 mmol/L, from the information and technology (IT) departments of these institutions. The study period was from January 2016 to July 2019. Patients under 18 years of age, those who were treated in the emergency room (ER) and then discharged, those with < 3 serum sodium measurements during hospitalization, and those with the second serum sodium measurement collected ≥ 30 hours after the initial measurement were excluded from the study.

Definition of Overcorrection of Hyponatremia: Overcorrection of hyponatremia was defined as an increase in serum sodium level > 8 mmol/L/24 hours or > 18 mmol/L/48 hours of admission. To allow comparison with other studies, the frequency of overcorrection was also assessed using the criterion of an increase in serum sodium level of > 10 mmol/L/24 hours, while maintaining the same criterion of > 18 mmol/L/48 hours.

Definition of Admission Serum Sodium, 24-hour Serum Sodium, and 48-hour Serum Sodium: Admission serum sodium was defined as the first serum sodium measurement obtained upon hospital admission, typically in the ER. Because this was an observational study, serum sodium levels were not always measured exactly 24 or 48 hours after admission. A deviation of up to 4 hours before or after these time-points was considered acceptable. If no serum sodium measurement was available within this time window, the 24- and 48-hour values were estimated based on the rate of change in serum sodium (mmol/L/h), using the available values. If a patient experienced a rapid increase in serum sodium (> 8 mmol/L) during the first 24 hours but had a subsequent decline such that the net change at 24 hours was < 8 mmol/L, the case was still classified as overcorrection. In these instances, however, specific interventions (e.g., discontinuation of hypertonic saline or administration of free water/hypotonic fluids) were undertaken to lower the serum sodium.

Sample Size Calculation: Given the scarcity of studies assessing the frequency of severe hyponatremia overcorrection in our region, we conducted a pilot study in one of the institutions. Starting from a list of cases of severe hyponatremia provided by the IT department of one institution, we analyzed a random sample of 50 patients, and found that the frequency of overcorrection was 32%. This percentage was then used for sample size calculation, which was performed using the free online tool Open Epi (https://www.openepi.com/SampleSize/SSPropor.htm). Assuming an infinite population, a frequency of overcorrection of 32%, and a margin of error of 5%, a minimum of 335 patients would need to be analyzed. Anticipating that some of the screened patients would be excluded, the medical records of approximately 485 patients were selected for review.

Ethical Aspects: The research project was submitted to and approved by the Institutional Review Board (IRB) of one of the institutions under the protocol number of 3.474.662 and we obtained a letter of agreement from the other collaborating institution. As this was a retrospective study involving the analysis of medical records, the IRB waived informed consent.

Data Collection: The case report form included demographic data, date of admission, date of hospital discharge or death, hospital disposition (intensive care unit or ward), whether there was an evaluation with a nephrologist for hyponatremia, as well as information about pre-existing conditions such as systemic arterial hypertension, diabetes mellitus (DM), cancer, chronic liver disease, chronic kidney disease (CKD), epilepsy or seizures, stroke, congestive heart failure (CHF), chronic obstructive pulmonary disease (COPD), as well as depression, bipolar mood disorder, schizophrenia, dementia, malnutrition, and history of alcoholism. Medications taken regularly were listed, provided they belonged to the following classes: diuretics, angiotensin-converting enzyme inhibitors (ACEIs) or angiotensin receptor blockers (ARBs), antidepressants, nonsteroidal anti-inflammatory drugs (NSAIDs), anticonvulsants, opioids, aldosterone antagonists, and cyclophosphamide. For the etiological investigation of hyponatremia, reports from chest and head computer tomography (CT) scans and head magnetic resonance imaging (MRI) were analyzed. As part of the etiological investigation, thyroid hormone levels and serum cortisol levels were collected if assessed at any time during the patient’s hospitalization. All serum sodium measurements within the first 48 hours were evaluated, including the dates and times of each measurement. In some cases, serum sodium measurements from the following 24 hours (total collection time of 72 hours) were also assessed to estimate the 48-hour sodium level. All medical orders written during the first 48 hours of admission were reviewed, searching for prescriptions for 0.9% NaCl, hypertonic saline (at 3% or another concentration > 0.9%), and dextrose solutions. Additionally, the administration of potassium was also reviewed, as well as whether there was a prescription for water restriction, dietary modification to a high-protein diet, and the administration of furosemide. The first solution infused in the ER, as well as the volume administered, was also recorded; however, it was not possible to confirm whether the entire prescribed volume (or only part of it) was administered. Data collection was carried out by the principal investigator (PI) and three medical students (APB, DRM, and DHLS) trained by the PI. Data regarding the cause of death was collected by two attending nephrologists (the PI and MGMG). All collected data were entered exclusively by the PI into a Microsoft Excel database and then transferred to SPSS for statistical analysis.

Statistical Analysis: Normally distributed continuous variables were summarized using mean and standard deviation, while non-normally distributed variables were summarized using median and interquartile range. Categorical variables were summarized with absolute and relative frequencies. Patients were stratified according to the presence or absence of hyponatremia overcorrection. The comparison of quantitative variables between the two groups was performed using the Student’s t-test, and categorical variables were compared using the chi-square test. To compare mean serum sodium at admission and at 24 hours within the same group, we used the paired t-test. To assess if there was a trend between the volume of 0.9% NaCl administered in the ER and the magnitude of serum sodium increase at 24 hours, we used the Jonckheere-Terpstra trend test. Multiple logistic regression was employed to identify independent predictors of hyponatremia overcorrection and mortality. In addition, we calculated propensity score-adjusted ORs for mortality. We used independent predictors of hyponatremia overcorrection to create propensity scores. Patients in the overcorrection group received weights equal to the inverse of their propensity scores whereas the remaining patients received weights equal to the inverse of (1 – propensity score). Finally, we used the weighted dataset to run a logistic regression with mortality as the outcome and hyponatremia overcorrection as the predictor. All analyses were conducted using the SPSS software for Windows version 20. A p-value < 0.05 in the final analyses was considered statistically significant.

## Results

### Description of the Sample (N = 362) and Frequency of Hyponatremia Overcorrection

**Figure 1.**
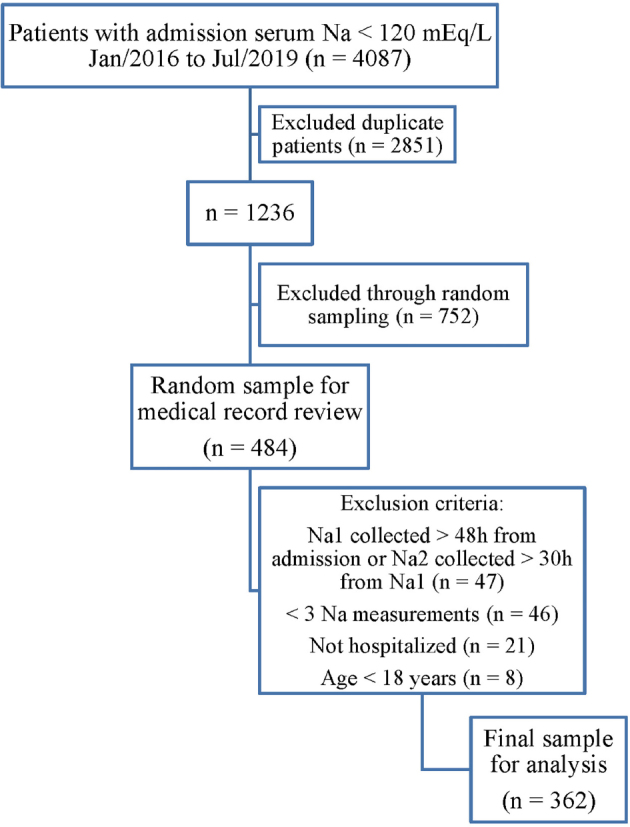
Flow chart describing the sampling process.

The sampling strategy is shown in Figure 1. The final sample consisted of 362 adult patients admitted with severe hyponatremia. The average age was 75.6 ± 13.6 years, and 65.5% were women. Hypertension (82.3%) and DM (76.2%) were the most prevalent comorbidities, and most patients (81.8%) were admitted to the intensive care unit (ICU), highlighting the clinical severity of the cases. The overall mortality was 26.2%.

Notes – Initially, all patients with records of admission serum Na < 120 mmol/L were screened from the electronic medical records of two tertiary hospitals in the period from January 2016 to July 2019. After excluding duplicate patient records, 1236 patients remained. Subsequently, we performed a random sample and reduced the sample size to 484 patients, a number well above the calculated minimum of 335 patients required for the final sample. We then reviewed the records and applied exclusion criteria: 47 patients were excluded because “Na1” (first serum sodium measurement after admission; admission sodium was referred to as “Na0”) was collected > 48 hours after admission, or “Na2” (second serum sodium measurement after admission) was collected > 30 hours after “Na1.” Additionally, 46 patients were excluded for having fewer than three serum sodium measurements during hospitalization. Twenty-one patients were excluded for not being hospitalized, and eight for being under 18 years of age. The final sample of 362 patients was composed of adult in-patients with severe hyponatremia, with three or more serum sodium measurements during admission within an interval that allowed the detection of overcorrection.

**Figure 2.**
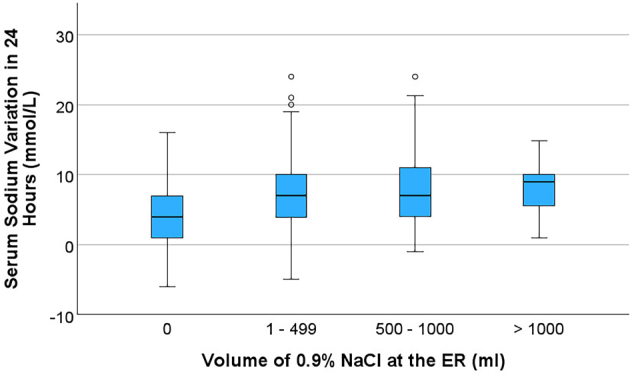
Serum sodium variation in 24 hours and volume of 0.9% NaCl administered at the ER.

Upon ER arrival, the mean admission serum sodium was 113.3 ± 4.9 mmol/L, ranging from 94 to 119 mmol/L (Figure S1). Most patients (73.2%) received 0.9% NaCl in the ER, with an average volume of 494.7 ± 392.8 mL. Only 6 patients received 3% NaCl or another hypertonic solution in the ER. There was a statistically significant correlation between the volume of 0.9% NaCl administered in the ER and the serum sodium variation in 24 hours (p for trend < 0.001) (Figure 2). In the first 24 hours, the average change in serum sodium was an increase of 6.65 ± 5.31 mmol/L, ranging from a decrease of 6 mmol/L to an increase of 24 mmol/L. Based on serum sodium variation in the first 24 hours, we stratified the evolution of natremia into six categories: decrease in serum sodium in 7.2%; no change in 3.9%; insufficient serum sodium increase (1.0 to 3.9 mmol/L) in 18.1%; ideal serum sodium increase (4.0 to 8.0 mmol/L) 31.6%; overcorrection in 35.5% (increase > 8 mmol/L in 24 hours); and overcorrection with successful re-dilution so that the difference between the sodium at the end of 24 hours and admission sodium was < 8 mmol/L in 3.3% (Figure S2).

The incidence of overcorrection considering the 24-hour (> 8 mmol/L) and the 48-hour (> 18 mmol/L) criteria was 38.7% (Table S1). The incidence of overcorrection decreased to 23% when the 24-hour criterion was changed from > 8 to > 10 mmol/L, while keeping the 48-hour criterion constant (> 18 mmol/L). This was done solely to compare our data with that of others. All subsequent analyses were done considering the 24-hour criterion of > 8 mmol/L.

There was only one case of ODS (0.28%).

### Description of the Sample Stratified by Hyponatremia Overcorrection

Table 1 shows the demographic, clinical, and outcome data of the patients stratified by presence or absence of hyponatremia overcorrection. Notable findings in the overcorrection group were younger age, lower admission serum sodium, fewer cancer cases, less furosemide use, lower mortality, and shorter hospital stay; overcorrected patients used more 0.9% NaCl, more D5W, had more hypokalemia, and used more potassium chloride (KCl).

**Table 1:** Demographic, clinical and outcome data of 362 patients admitted with severe hyponatremia stratified for the presence or absence of overcorrection of hyponatremia

Variables	Overcorrection		p value
	No (n = 222)	Yes (n = 140)	
Age	76.81 ± 13.27	73.57 ± 13.85	**0.027**
Women	144 (64.9%)	93 (66.4%)	0.848
Admission serum sodium (mmol/L)	114.23 ± 4.37	111.85 ± 5.40	**<0.001**
Comorbidities, n (%)			
Hypertension	180 (81.1%)	118 (84.3%)	0.524
Diabetes	78 (35.1%)	48 (34.3%)	0.959
Cancer	56 (25.2%)	22 (15.7%)	**0.044**
Stroke	34 (15.3%)	31 (22.1%)	0.132
Heart failure	37 (16.7%)	19 (13.6%)	0.520
Dementia	28 (12.6%)	20 (14.3%)	0.766
Epilepsy/seizure	15 (6.8%)	16 (11.4%)	0.176
CKD	18 (8.1%)	12 (8.6%)	1.000
Depression	15 (6.8%)	12 (8.6%)	0.664
COPD	12 (5.4%)	3 (2.1%)	0.213
Alcoholism	8 (3.6%)	2 (1.4%)	0.368
Schizophrenia	3 (1.4%)	1 (0.7%)	0.961
Malnutrition	3 (1.4%)	1 (0.7%)	0.961
Chronic liver disease	2 (0.9%)	0 (0.0%)	0.690
Medications, n (%)			
ARB/ACE inhibitor	95 (42.8%)	70 (50.0%)	0.218
Diuretics	69 (31.1%)	53 (37.9%)	0.225
Antidepressant	33 (14.9%)	15 (10.7%)	0.330
Anticonvulsant	22 (9.9%)	17 (12.1%)	0.622
Opioid	17 (7.7%)	6 (4.3%)	0.285
Aldosterone antagonist	16 (7.2%)	7 (5.0%)	0.537
NSAID	11 (5.0%)	3 (2.1%)	0.284
Cyclophosphamide	2 (0.9%)	0 (0.0%)	0.690
ICU, n (%)	180 (81.1%)	116 (82.9%)	0.775
Death, n (%)	72 (32.4%)	23 (16.4%)	**0.001**
ODS, n (%)	0 (0.0%)	1 (0.7%)	0.387
Length of stay, median [IQR]	11 [7–16.5]	9 [6–14]	**0.024**
Inpatient treatment, n (%)			
3% hypertonic saline	49 (22.0%)	34 (24.2%)	0.719
Other hypertonic saline	37 (16.6%)	21 (15.0%)	0.784
0.9% NaCl	193 (86.9%)	134 (95.7%)	**0.010**
5% dextrose solution	24 (10.8%)	30 (21.4%)	**0.009**
Furosemide	98 (44.1%)	38 (27.1%)	**0.002**
Water restriction	49 (22.0%)	19 (13.5%)	0.091
Solute-rich diet	11 (4.8%)	4 (2.8%)	0.481
KCl	56 (25.2%)	51 (36.4%)	**0.031**
Volume of 0.9% NaCl at the ER, n (%)			
0 ml	72 (33.2%)	19 (13.7%)	**<0.001**
up to 499 ml	6 (2.8%)	6 (4.3%)	
500–1000 ml	136 (62.7%)	108 (77.7%)	
> 1000 ml	3 (1.4%)	6 (4.3%)	
Etiological investigation, n (%)			
Cortisol < 5 mcg/dL (n = 81)	2 (3.5%)	0 (0.0%)	0.885
TSH > 10 (n = 159)	15 (15.3%)	9 (14.8%)	1.000
Free T4 < 0.7 ng/dL (n = 157)	4 (4.2%)	2 (3.3%)	1.000
Urea > 40 mg/dL (n = 362)	91 (41%)	52 (37.1%)	0.536
Creatinine > 1.2 mg/dL (n = 362)	45 (20.3%)	28 (20.0%)	1.000
Potassium < 3.5 mmol/L (n = 362)	20 (9.0%)	34 (24.3%)	**<0.001**
Low uric acid (n = 32)	9 (52.9%)	10 (66.7%)	0.668
Urinary sodium < 20 (n = 39)	3 (10.7%)	2 (18.2%)	0.924
Urinary sodium < 40 (n = 39)	7 (25.0%)	5 (45.5%)	0.390

Abbreviations – For the variable age, n = 361. For the variable volume of 0.9% NaCl, n = 356. Continuous variables are expressed as mean and standard deviation. CKD: chronic kidney disease; COPD: chronic obstructive pulmonary disease; ARB: angiotensin receptor blocker; ACE: angiotensin-converting enzyme; NSAID: non-steroidal anti-inflammatory drug; ICU: intensive care unit; ODS: osmotic demyelination syndrome; IQR: interquartile range; NaCl: sodium chloride; KCl: potassium chloride; ER: emergency room; TSH: thyroid-stimulating hormone.

**Figure 3.**
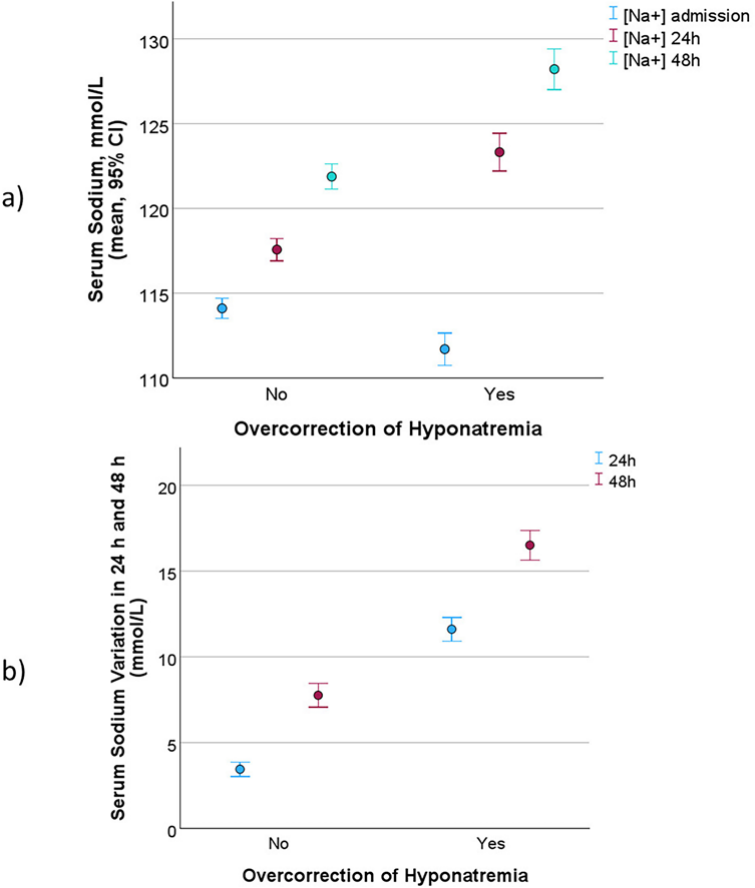
Evolution of serum sodium in the first 48 hours. (a) Serum sodium at admission, 24 hours, and 48 hours stratified by overcorrection of hyponatremia; (b) serum sodium variation at 24 hours and at 48 hours stratified by overcorrection of hyponatremia.

Figure 3a shows serum sodium results. First, the mean admission serum sodium was significantly lower in the overcorrection group (111.9 mmol/L compared to 114.2 mmol/L, p < 0.001). Second, the mean serum sodium levels at 24 and at 48 hours were significantly higher in the overcorrection group (24 hours: 123.6 compared to 117.6 mmol/L, p < 0.001; 48 hours: 128.2 compared to 121.9 mmol/L, p < 0.001). It is noteworthy that the mean sodium level at 24 hours in the overcorrection group is numerically higher than the mean sodium level at 48 hours in the group that did not overcorrect. Figure 3b shows that the mean increase in serum sodium at 24 hours in the overcorrection group was 11.8 mmol/L compared to only 3.4 mmol/L in the group that did not overcorrect (p < 0.001); the mean increase in serum sodium at 48 hours was also significantly higher in the overcorrection group (16.5 mmol/L compared to 7.8 mmol/L, p < 0.001).

**Figure 4.**
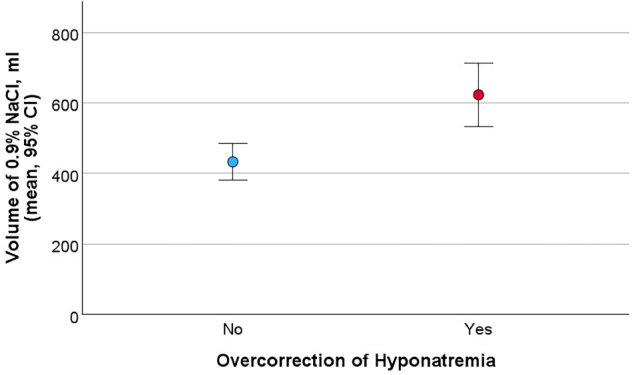
Volume of 0.9% NaCl Administered at the Emergency Room Stratified by Overcorrection of Hyponatremia.

Figure 4 shows that the overcorrection group received, on average, a significantly larger volume of 0.9% NaCl in the ER compared to those who did not overcorrect (623.02 ± 535.39 vs. 433.18 ± 387.34 mL, p < 0.001).

### Independent Predictors of Hyponatremia Overcorrection

To identify independent predictors of hyponatremia overcorrection, we used multiple logistic regression. The model included the variables associated with the outcome in the univariate analyses. Table 2 displays the results of two models. In the first model, the continuous variables age, admission serum sodium and volume of 0.9% NaCl in the ER were stratified using their mean value as the cutoff point. In the second model, these three variables were evaluated in their continuous format.

**Table 2:** Independent predictors of overcorrection of hyponatremia

Independent variables	OR	95% CI	p value
Model 1			
0.9% NaCl ≥ 500 ml in ER	2.10	1.22–3.61	0.007
Admission serum sodium ≤ 113 mmol/L	2.40	1.52–3.80	<0.001
Age ≤ 75 years	1.96	1.22–3.13	0.005
Cancer	0.45	0.25–0.82	0.008
Furosemide use	0.52	0.32–0.84	0.008
Model 2			
0.9% NaCl (each 100 ml) in the ER	1.10	1.04–1.17	0.002
Admission serum sodium	0.91	0.87–0.95	<0.001
Age	0.98	0.97–0.99	0.035
Cancer	0.55	0.31–0.99	0.047
Furosemide use	0.46	0.28–0.76	0.002

Abbreviations – NaCl: sodium chloride.

Notes – In Model 1, continuous variables were categorized using the mean as the cutoff point. In Model 2, continuous variables were treated as such. For the variable related to the volume of NaCl administered in the ER to be more clinically relevant, we multiplied the betas by 100 and then took the natural logarithm, so that the odds ratios expressed the risk for every 100 ml of solution.

Multivariate analysis revealed that younger age, a lower admission serum sodium and a higher volume of 0.9% NaCl administered in the ER were associated with higher odds of overcorrection. Conversely, a diagnosis of cancer and the use of furosemide were associated with lower odds of overcorrection.

### Impact of Hyponatremia Overcorrection

**Figure 5.**
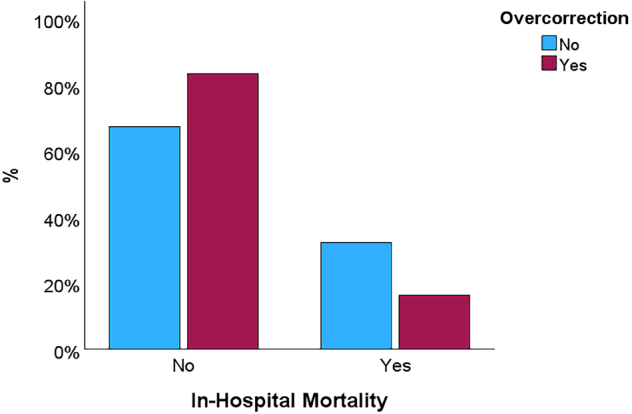
In-hospital mortality stratified by hyponatremia overcorrection.

Unadjusted in-hospital mortality was significantly lower in patients who experienced hyponatremia overcorrection (16.4% versus 32.4%, p = 0.001) (Figure 5). Patients who died had significantly lower serum sodium levels at admission (112.30 ± 5.8 mmol/L vs. 113.67 ± 4.5 mmol/L, p = 0.039), at 24 hours (117.58 ± 5.7 mmol/L vs. 120.77 ± 6.2 mmol/L; p < 0.001), and at 48 hours (122.04 ± 6.9 mmol/L vs. 125.16 ± 6.6 mmol/L, p < 0.001) (Figure S3). This translated into a significantly lower increase in serum sodium in those who died compared to survivors, both at 24 hours (5.27 ± 4.5 mmol/L vs 7.1 ± 5.5 mmol/L, p = 0.002) and at 48 hours (9. 47 ± 6.0 mmol/L vs 11.72 ± 6.6 mmol/L, p = 0.016) (Figure S3).

The adjusted association between hyponatremia overcorrection and lower mortality was assessed with multivariate analysis. To select variables for the multivariate model, we initially performed univariate analyses stratifying the cohort by mortality (Table S2). The variables significantly associated with increased mortality in the univariate analyses were older age, male gender, a lower serum sodium concentration on admission, a diagnosis of cancer, furosemide use during hospitalization, and higher levels of urea and creatinine on admission. Overcorrection of hyponatremia, use of ACE-i/ARB at home, and water restriction during hospitalization were associated with decreased mortality. Alcoholism, chronic liver disease, ICU admission, and longer hospital length of stay were marginally associated with increased mortality. A diagnosis of hypertension, use of diuretics at home, hypokalemia, hypouricemia, and a low urinary sodium were marginally associated with decreased mortality. Since there were only two patients with chronic liver disease and too much data were missing for uric acid and urinary sodium, these variables were excluded from further analyses. All other variables were included in a stepwise backward multivariate logistic regression. As shown in Table 3, hyponatremia overcorrection was associated with a 64% reduction in the odds of mortality. Use of ACE-i/ARB at home and water restriction in the hospital were also associated with reduced mortality. Older age, male gender, cancer, a lower admission serum sodium, a higher admission serum creatinine, and use of furosemide during hospitalization were associated with higher odds of in-hospital mortality. Finally, we used baseline predictors of overcorrection to create propensity scores and then ran a propensity score-weighted logistic regression analysis with mortality as the outcome and overcorrection as the predictor. In this analysis, hyponatremia overcorrection was still associated with a 45% reduction in the odds of mortality (OR 0.548, 95% CI 0.389 to 0.772, p < 0.001).

**Table 3:** Independent predictors of death

Independent variables	OR	95% CI	p value
Overcorrection of hyponatremia	0.364	0.196–0.675	0.001
Age	1.041	1.017–1.066	<0.001
Male gender	2.088	1.181–3.691	0.011
ARB/ACE inhibitor (home)	0.382	0.213–0.684	0.045
Cancer	2.370	1.268–4.430	0.007
Admission serum sodium ≤ 113 mmol/L	2.166	1.218–3.851	0.008
Admission serum creatinine > 1.2 mg/dL	2.810	1.482–5.329	0.002
Furosemide (hospital)	1.788	1.012–3.157	0.001
Water restriction (hospital)	0.186	0.073–0.473	<0.001

Abbreviations – ARB: angiotensin receptor blocker; ACE: angiotensin-converting enzyme.

Notes – Variables inserted in the initial step: age, gender, hypertension, cancer, alcoholism, ARB/ACE inhibitor (home), diuretic (home), furosemide (hospital), water restriction, (hospital), admission serum creatinine > 1.2 mg/dL, admission serum urea > 40 mg/dL, overcorrection of hyponatremia, admission serum potassium < 3.5 mmol/L, admission serum sodium ≤ 113 mmol/L, ICU admission, length of hospital stay.

### Cause of Death

To evaluate whether the rate of correction of serum sodium had direct influence on mortality, we carefully reviewed the charts of all 95 patients who died. As shown in Table 4, sepsis was the leading cause of death (55.8%). Overlapping conditions were common; for instance, some patients with sepsis also had cancer or heart disease, although these comorbidities were not considered the primary cause of death. Similarly, cancer (15.8%) was assigned as the cause of death when disease progression was identified as the main factor. Aspiration of gastric contents (13.7%) was considered as a potential cause of hyponatremic death, identified through active chart review; other comorbidities such as cancer or infection might have been present but were secondary. Cardiac-related deaths (7.4%) included acute decompensation of congestive heart failure, cardiogenic shock, and complications from cardiac surgery. Other causes included stroke (4.2%), bleeding (2.1%), and ODS (1.1%). All 13 deaths from aspiration of gastric contents were reviewed in detail; 11 (15.3%) occurred in those who did not overcorrect whereas only 2 (8.7%) occurred in the overcorrection group. In 8 of these 13 deaths, aspiration of gastric contents could not be linked to hyponatremia (because of palliative care initiation, significant improvement in hyponatremia, or other causes of aspiration). Therefore, 5 deaths were considered potentially linked to severe hyponatremia, 4 (5.6%) of which occurred in the non-overcorrection group and 1 (4.3%) occurred in the overcorrection group. We found one case of ODS (1.1%), which occurred in the overcorrected group (Table 4).

**Table 4:** Causes of death stratified by overcorrection of hyponatremia

Causes of death	Total (n = 95)	Overcorrection	
		No (n = 72)	Yes (n = 23)
Sepsis	53 (55.8%)	37 (51.4%)	16 (69.6%)
Cancer	15 (15.8%)	14 (19.4%)	1 (4.3%)
Aspiration of gastric contents	13 (13.7%)	11 (15.3%)	2 (8.7%)
Possibly related to hyponatremia	5 (5.3%)	4 (5.6%)	1 (4.3%)
Patients on palliative care	3 (3.2%)	2 (2.8%)	1 (4.3%)
Probably not related to hyponatremia	5 (5.3%)	5 (6.9%)	0 (0.0%)
Cardiac	7 (7.4%)	7 (9.7%)	0 (0.0%)
Stroke	4 (4.2%)	2 (2.8%)	2 (8.7%)
Bleeding	2 (2.1%)	1 (1.4%)	1 (4.3%)
ODS	1 (1.1%)	0 (0.0%)	1 (4.3%)

Abbreviations – ODS: osmotic demyelination syndrome.

Notes – Of the 13 cases of aspiration of gastric contents, 3 were on palliative care and 5 did not have severe hyponatremia or had other reasons for encephalopathy at the time of aspiration of gastric contents. Therefore, only 5 cases were considered possibly linked to severe hyponatremia. Of these, 4 did not overcorrect and 1 overcorrected the hyponatremia.

## Discussion

### General Statement

The frequency of severe hyponatremia overcorrection in patients admitted to two Brazilian tertiary hospitals was 38.7%. Greater severity of hyponatremia, higher volumes of 0.9% NaCl infusion in the ER, and lower age increased the risk of overcorrection. The diagnosis of cancer and the use of furosemide were associated with a lower risk of overcorrection. Finally, overcorrection of hyponatremia was associated with a shorter hospital stay and lower odds of in-hospital mortality.

### Frequency of Overcorrection

The frequency of hyponatremia overcorrection has been studied by other groups. A multicenter study conducted in Pennsylvania evaluated 1490 patients admitted with severe hyponatremia and found frequencies of overcorrection almost identical to our study: 41% when using the > 8 mmol/L/24 hours criterion and 26% with the > 10 mmol/L/24 hours criterion^16^. Another study, also involving patients with severe hyponatremia, observed a frequency of overcorrection of 27.9% using the > 10 mmol/L/24 hours criterion^24^. Therefore, our results are consistent with what has been reported in the literature and suggest that hyponatremia overcorrection is a global phenomenon, not limited to the centers we studied. To the best of our knowledge, this is the first study on this topic in Latin America.

### Risk Factors for Overcorrection

Even within this sample of patients with severe hyponatremia, the lower the admission serum sodium, the higher the odds of overcorrection. The mean admission serum sodium was significantly lower in the overcorrection group (111.9 ± 5.4 mmol/L versus 114.2 ± 4.4 mmol/L, p < 0.001), and an admission sodium ≤ 113 mmol/L was associated with 2.4 times higher odds of overcorrection (p < 0.001). Previous studies also identified hyponatremia severity as a risk factor for overcorrection. An American study published in 2007 evaluated 62 patients treated with hypertonic saline and found that the mean admission serum sodium level was lower in those who experienced overcorrection compared to those who did not (111.9 ± 1.5 mmol/L versus 118.5 ± 0.8 mmol/L, p = 0.002)^25^. A retrospective, single-center study that analyzed 56 patients with sodium levels < 125 mmol/L found that the admission sodium level had a significant impact on overcorrection. The authors observed that each 1-mmol/L increase in the initial sodium value reduced the risk of overcorrection by 16% (p = 0.037)^26^. In our study, each 1-mmol/L increase in admission serum sodium reduced the odds of overcorrection by 9% (p < 0.001).

The ideal therapy for patients with severe hyponatremia remains controversial. Some physicians still use formulas to guide the use of continuous infusions of NaCl solutions. However, due to the high frequency of overcorrection when applying this strategy, several experts, including the American and European guidelines, recommend the use of small, fixed boluses (100–150 mL) of hypertonic saline for cases of severe and acute hyponatremia^27^. In our study, only 1.6% of patients were treated with hypertonic saline at the ER. One possible explanation could be the presence of mild symptoms that did not justify the use of hypertonic solution. However, we cannot rule out the possibility that limited familiarity with hypertonic solutions or with current guidelines, as well as a low perception of the risk of overcorrection, may have influenced therapeutic decision. Most of our patients were treated with 0.9% NaCl in the ER (73.2%). Among them, 68.5% received 0.9% NaCl volumes between 500 and 1000 ml. The administration of 0.9% NaCl volumes > 500 ml in the ER, a common treatment for hypovolemic patients, doubled the risk of overcorrection. For every additional 100 mL of 0.9% NaCl infused in the ER, the risk of overcorrection increased by 10%.

We observed an inverse relationship between age and overcorrection. For each additional year in age, the odds of overcorrection decreased by 2%. The average age of the patients in our sample was 75.6 ± 13.6 years, but the group that was overcorrected had a younger average age (73.57 ± 13.85 vs. 76.81 ± 13.27 years, p = 0.027). Patients above the mean age had nearly twice the odds of overcorrection. This inverse association between age and overcorrection has also been described by other authors [4, 5, 8], but the cause of this association is not clear.

We observed two protective factors against overcorrection: having cancer and using furosemide. Patients with cancer had a lower risk of overcorrection, possibly because patients with syndrome of inappropriate antidiuresis (SIAD) are protected from autocorrection due to constantly elevated ADH levels. We also noticed that, although furosemide increases renal water clearance, patients who used furosemide in our study had lower odds of overcorrection. Our hypothesis is that furosemide use acted as a proxy for diagnoses less prone to autocorrection, such as SIAD or hypervolemic hyponatremia.

### Impact of Overcorrection

Patients who experienced hyponatremia over­correction had a median hospital stay 2 days shorter than those who did not overcorrect (9 versus 11 days, p = 0.024). This is in line with the work of Seethapathy et al.^20^ showing a significantly shorter hospital stay for patients who corrected by > 10 mmol/L/day compared to the group that corrected by < 6 mmol/L/day. In further agreement with the data by Seethapathy et al.^20^, we also found a lower in-hospital mortality in patients who experienced overcorrection compared to those who did not overcorrect (16.4 vs. 32.4%; p = 0.001). The association between hyponatremia overcorrection and lower mortality persisted after adjusting for relevant covariates in multivariable analyses and in propensity score-weighted analyses. A recent metanalysis by Ayus et al.^22^ summarizing sixteen cohort studies involving 11,811 patients with severe hyponatremia indicated that rapid correction was associated with fewer in-hospital deaths compared with slower correction; moreover, rapid correction was not associated with a statistically significant increased risk of ODS.

### Hyponatremia as a Direct Cause of Death?

Although acute and extreme hyponatremia may directly cause death by brain herniation if inadequately treated, this is not how patients died in our cohort. Whether less extreme hyponatremia with a more chronic course can contribute to mortality is controversial. After carefully reviewing the charts of all 95 patients who died in our cohort, we found 5 (5.3%) cases of aspiration of gastric contents that could have been precipitated by hyponatremic encephalopathy; 4 in the non-overcorrection group (5.6%) and 1 in the overcorrection group (4.3%). Therefore, we posit that the association between overcorrection and lower mortality is non causal. For example, the reported association between the severity of hyponatremia and higher mortality in patients with heart failure and cirrhosis, might simply be indicating that hyponatremia is a marker for the degree of hemodynamic imbalance caused by the underlying disease. Likewise, the association between overcorrection and lower mortality in observational studies may not be causal but due to confounding. Instead of showing that a faster correction of hyponatremia is protecting patients from hyponatremic deaths, overcorrection might be simply identifying a group of patients who *are prone to* correct faster due to the nature of their underlying disease. One of the main causes of hyponatremia overcorrection is the treatment of hypovolemia, raising the question whether overcorrection may actually identify patients with hypovolemia—who could be less severely ill and therefore less likely to die. On the other hand, the absence of overcorrection might reflect patients with constantly elevated ADH levels from cancer-induced SIAD, cirrhosis, or heart failure. Although we adjusted for these variables in multivariate analyses, residual confounding cannot be excluded.

### Limitations of The Study

This study has several limitations, most of them related to its observational nature. First, it was necessary to estimate the serum sodium values at 24 and 48 hours in some patients since not all had sodium measurements at those exact times; a similar strategy was recently used by Seethapathy et al.^20^. Second, as the exact volume of saline infused in the ER could not be determined, we relied on the volumes prescribed and verified by nursing staff, without certainty that the entire prescribed volume was indeed infused. The timing of furosemide use was also not collected. We suspect that furosemide use served as a proxy for cases of SIAD and hypervolemia, which would explain its protective effect against overcorrection, as in these clinical conditions, overcorrection is less likely due to persistently elevated ADH levels. Third, data on patient symptoms, precise volume status, and the etiological diagnosis of hyponatremia were also not systematically available in the records, which is an important limitation as these variables guide the management of hyponatremia in daily practice. Fourth, we did not have serum glucose levels measured concomitantly with serum sodium on admission to assess hyperosmolar hyponatremia. Fifth, despite controlling for relevant covariates, the association between overcorrection and lower mortality could still be due to residual confounding. Upon detailed review of the medical records of all deceased patients, we could not establish that hyponatremia was causally related to excess deaths in the group that corrected more slowly.

## Conclusions

Overcorrection of severe hyponatremia was common in the two Brazilian centers studied, especially in patients who were younger, had lower admission serum sodium, and received more saline at the ER; conversely, having cancer and using furosemide during hospitalization reduced the odds of overcorrection. In agreement with recent observational studies, ODS was rare, and mortality was significantly lower in patients who experienced overcorrection. It is possible that hyponatremia overcorrection may simply serve as a proxy for rapidly reversible causes of hyponatremia, ultimately reflecting underlying diagnoses less likely to cause death. Therefore, randomized clinical trials are needed before the current safeguards established by hyponatremia guidelines are abandoned. These trials should address some of the limitations of our work and include a formal assessment of symptoms and volume status to guide sodium correction.

## Data Availability

All data generated or analyzed during this study are included in this article and its supplementary material files. Further enquiries can be directed to the corresponding author. The dataset that supports the findings of this study is not publicly available because it contains information that could compromise the privacy of research participants but is available from PNR upon reasonable request.

## Supplementary Material

The following online material is available for this article:

Figure S1 - Distribution of serum sodium at admission.

Figure S2 - Categorization of the evolution of serum sodium in the first 24 hours.

Figure S3 - Serum sodium at admission, 24 hours, and 48 hours stratified by in-hospital mortality.

Table S1 - Frequency of overcorrection of serum sodium in 362 patients admitted with severe hyponatremia.

Table S2 - Demographic, clinical and outcome data of 362 patients admitted with severe hyponatremia stratified for mortality.
