# Supplementary material for: Overcorrection of severe hyponatremia, osmotic demyelination syndrome, and mortality: insights from two Brazilian centers
Source: J Bras Nefrol. 2026 Jan 23;48(1):e20250161. doi: 10.1590/2175-8239-JBN-2025-0161en (PMC12854713; doi:10.1590/2175-8239-JBN-2025-0161en)
Supplement: Table S1 - [file 2175-8239-jbn-48-1-e20250161-suppl4.pdf]

Braz. J. Nephrol.
https://doi.org/10.1590/2175-8239-JBN-2025-0161en

Supplementary Material to "Overcorrection of severe hyponatremia, osmotic demyelination syndrome, and mortality: insights from two Brazilian centers"

Table S1 - Frequency of overcorrection of serum sodium in 362 patients admitted with severe hyponatremia.

Overcorrection criteria adopted	N (%)
Serum [Na^+] variation > 8 mmol/L/24 h	100 (27.6%)
Serum [Na^+] variation > 18 mmol/L/48 h	5 (1.4%)
Serum [Na^+] variation > 8 mmol/L/24 h e > 18 mmol/L/48 h	35 (9.7%)
Total	140 (38.7%)
