# Supplementary material for: Overcorrection of severe hyponatremia, osmotic demyelination syndrome, and mortality: insights from two Brazilian centers
Source: J Bras Nefrol. 2026 Jan 23;48(1):e20250161. doi: 10.1590/2175-8239-JBN-2025-0161en (PMC12854713; doi:10.1590/2175-8239-JBN-2025-0161en)
Supplement: Table S2 - [file 2175-8239-jbn-48-1-e20250161-suppl5.pdf]

Braz. J. Nephrol.
https://doi.org/10.1590/2175-8239-JBN-2025-0161en

Supplementary Material to "Overcorrection of severe hyponatremia, osmotic demyelination syndrome, and mortality: insights from two Brazilian centers"

Table S2 - Demographic, clinical and outcome data of 362 patients admitted with severe hyponatremia stratified for mortality.

Variables	Death		p value
	No (n = 267)	Yes (n = 95)	
Age	74.66 ± 14.30	78.06 ± 14.08	**0.036**
Women	188 (70.4%)	49 (51.6%)	**0.001**
Admission serum sodium (mmol/L)	113.67 ± 4.53	112.30 ± 5.81	**0.039**
24-hour serum sodium (mmol/L) (n = 361)	120.77 ± 6.16	117.58 ± 5.69	**< 0.001**
24-hour delta sodium (mmol/L) (n = 361)	7.10 ± 5.50	5.27 ± 4.51	**0.002**
48-hour serum sodium (mmol/L) (n = 330)	125.16 ± 6.56	122.04 ± 6.86	**< 0.001**
48-hour delta sodium (mmol/L) (n = 330)	11.72 ± 6.61	9.74 ± 6.03	**0.016**
Overcorrection of hyponatremia	117 (43.8%)	23 (24.2%)	**0.001**
Comorbidities, n (%)			
Hypertension	226 (84.6%)	72 (75.8%)	**0.074**
Diabetes	94 (35.2%)	32 (33.7%)	0.887
Cancer	45 (16.9%)	33 (34.7%)	**< 0.001**
Stroke	44 (16.5%)	21 (22.1%)	0.284
Heart failure	40 (15.0%)	16 (16.8%)	0.791
Dementia	32 (12.0%)	16 (16.8%)	0.306
Epilepsy/seizure	12 (4.5%)	1 (1.1%)	0.197
CKD	19 (7.1%)	11 (11.6%)	0.255
Depression	22 (8.2%)	5 (5.3%)	0.471
COPD	12 (4.5%)	3 (3.2%)	0.768
Alcoholism	5 (1.9%)	5 (5.3%)	**0.136**
Schizophrenia	4 (1.5%)	0 (0.0%)	0.577
Malnutrition	2 (0.7%)	2 (2.1%)	0.282
Chronic liver disease	0 (0.0%)	2 (2.1%)	**0.068**
Medications, n (%)			
ARB / ACE inhibitor	139 (52.1%)	26 (27.4%)	**<0.001**

Variables	Death		p value
	No (n = 267)	Yes (n = 95)	
Diuretics	98 (36.7%)	24 (25.3%)	**0.057**
Antidepressant	39 (14.6%)	9 (9.5%)	0.223
Anticonvulsant	31 (11.6%)	8 (8.4%)	0.504
Opioid	15 (5.6%)	8 (8.5%)	0.458
Aldosterone antagonist	14 (5.2%)	9 (9.5%)	0.228
NSAID	10 (3.7%)	4 (4.2%)	0.766
Cyclophosphamide	1 (0.4%)	1 (1.1%)	0.457
ICU, n (%)	212 (79.4%)	84 (88.4%)	**0.072**
Length of stay, median [IQR]	10 [6 – 15]	11 [7 – 20]	**0.086**
Inpatient treatment, n (%)			
3% hypertonic saline	61 (22.8%)	22 (23.2%)	1.000
Other hypertonic saline	44 (16.5%)	14 (14.7%)	0.814
0.9% NaCl	242 (90.6%)	85 (89.5%)	0.899
5% dextrose solution	42 (15.7%)	12 (12.6%)	0.575
Furosemide	89 (33.3%)	47 (49.5%)	**0.008**
Water restriction	62 (23.2%)	7 (7.4%)	**0.001**
Solute-rich Diet	11 (4.1%)	4 (4.2%)	1.000
KCl	82 (30.7%)	25 (26.3%)	0.499
Volume of 0.9% NaCl at the ER, n (%)			
• 0 ml	64 (24.0%)	27 (28.4%)	0.751
• up to 499 ml	8 (3.0%)	4(4.2%)	
• 500 - 1000 ml	184 (68.9%)	60 (63.2%)	
• > 1000 ml	11 (4.1%)	4 (4.2%)	
Etiological investigation, n (%)			
Cortisol < 5 mcg/dL (n = 81)	2 (2.9%)	0 (0.0%)	1.000
TSH > 10 (n = 159)	18 (14.0%)	6 (20.0%)	0.403
Free T4 < 0.7 ng/dL (n = 157)	5 (3.9%)	1 (3.6%)	1.000
Urea > 40 mg/dL (n = 362)	89 (33.3%)	54 (56.8%)	**< 0.001**
Creatinine > 1.2 mg/dL (n = 362)	42 (15.7%)	31 (32.6%)	**< 0.001**
Potassium < 3.5 mmol/L (n = 362)	45 (16.9%)	9 (9.5%)	**0.117**
Low uric acid (n = 32)	17 (68.0%)	2 (28.6%)	**0.091**
Urinary sodium < 20 (n = 39)	5 (15.6%)	0 (0.0%)	0.563
Urinary sodium < 40 (n = 39)	12 (37.5%)	0 (0.0%)	**0.077**

Abbreviation - CKD = chronic kidney disease; COPD = chronic obstructive pulmonary disease; ARB = angiotensin receptor blocker; ACE = angiotensin-converting enzyme; NSAID = non-steroidal anti-inflammatory drug; ICU = intensive care unit; IQR = interquartile range; NaCl = sodium chloride; KCl = potassium chloride; ER = emergency

room; TSH = thyroid-stimulating hormone. Note - For the variable age, n = 361. For the variable volume of 0.9% NaCl, n = 356. Continuous variables are expressed as mean and standard deviation.
