# Supplementary material for: Overcorrection of severe hyponatremia, osmotic demyelination syndrome, and mortality: insights from two Brazilian centers
Source: J Bras Nefrol. 2026 Jan 23;48(1):e20250161. doi: 10.1590/2175-8239-JBN-2025-0161en (PMC12854713; doi:10.1590/2175-8239-JBN-2025-0161en)
Supplement: Figura S3 - [file 2175-8239-jbn-48-1-e20250161-suppl8.pdf]

Braz. J. Nephrol.
https://doi.org/10.1590/2175-8239-JBN-2025-0161pt

Material Suplementar para “Hipercorreção da hiponatremia grave, síndrome de desmielinização osmótica e mortalidade: percepções de dois centros brasileiros”

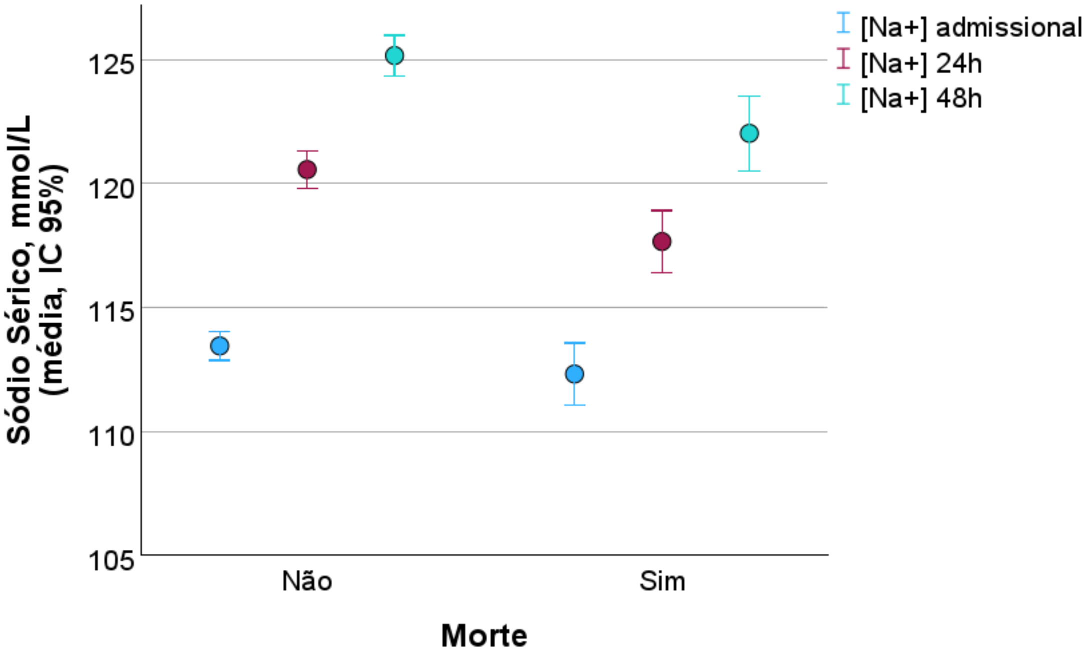

Figura S3 – Sódio sérico na admissão, em 24 horas e em 48 horas estratificado por mortalidade hospitalar.
