# Supplementary material for: Overcorrection of severe hyponatremia, osmotic demyelination syndrome, and mortality: insights from two Brazilian centers
Source: J Bras Nefrol. 2026 Jan 23;48(1):e20250161. doi: 10.1590/2175-8239-JBN-2025-0161en (PMC12854713; doi:10.1590/2175-8239-JBN-2025-0161en)
Supplement: Tabela S1 - [file 2175-8239-jbn-48-1-e20250161-suppl9.pdf]

Braz. J. Nephrol.
https://doi.org/10.1590/2175-8239-JBN-2025-0161pt

Material Suplementar para "Hipercorreção da hiponatremia grave, síndrome de desmielinização osmótica e mortalidade: percepções de dois centros brasileiros"

Tabela S1 - Frequência de Hipercorreção do Sódio Sérico em 362 Pacientes Internados com Hiponatremia Grave.

Critérios de hipercorreção adotados	N (%)
Variação do [Na+] sérico > 8 mmol/L/24 h	100 (27,6%)
Variação do [Na+] sérico > 18 mmol/L/48 h	5 (1,4%)
Variação do [Na+] sérico > 8 mmol/L/24 h e > 18 mmol/L/48 h	35 (9,7%)
Total	140 (38,7%)
