# Supplementary material for: Overcorrection of severe hyponatremia, osmotic demyelination syndrome, and mortality: insights from two Brazilian centers
Source: J Bras Nefrol. 2026 Jan 23;48(1):e20250161. doi: 10.1590/2175-8239-JBN-2025-0161en (PMC12854713; doi:10.1590/2175-8239-JBN-2025-0161en)
Supplement: Tabela S2 - [file 2175-8239-jbn-48-1-e20250161-suppl10.pdf]

Braz. J. Nephrol.
https://doi.org/10.1590/2175-8239-JBN-2025-0161pt

Material Suplementar para "Hipercorreção da hiponatremia grave, síndrome de desmielinização osmótica e mortalidade: percepções de dois centros brasileiros"

Tabela S2 - Dados Demográficos, Clínicos e de Desfecho de 362 Pacientes Internados com Hiponatremia Grave, Estratificados por Mortalidade.

Variáveis	Morte		Valor p
	Não (n = 267)	Sim (n = 95)	
Idade	74,66 ± 14,30	78,06 ± 14,08	**0,036**
Mulheres	188 (70,4%)	49 (51,6%)	**0,001**
Sódio sérico na admissão (mmol/L)	113,67 ± 4,53	112,30 ± 5,81	**0,039**
Sódio sérico em 24 horas (mmol/L) (n = 361)	120,77 ± 6,16	117,58 ± 5,69	**< 0,001**
Delta sódio em 24 horas (mmol/L) (n = 361)	7,10 ± 5,50	5,27 ± 4,51	**0,002**
Sódio sérico em 48 horas (mmol/L) (n = 330)	125,16 ± 6,56	122,04 ± 6,86	**< 0,001**
Delta sódio em 48 horas (mmol/L) (n = 330)	11,72 ± 6,61	9,74 ± 6,03	**0,016**
Hipercorreção da hiponatremia	117 (43,8%)	23 (24,2%)	**0,001**
Comorbidades, n (%)			
Hipertensão	226 (84,6%)	72 (75,8%)	**0,074**
Diabetes	94 (35,2%)	32 (33,7%)	0,887
Câncer	45 (16,9%)	33 (34,7%)	**< 0,001**
Acidente vascular cerebral	44 (16,5%)	21 (22,1%)	0,284
Insuficiência cardíaca	40 (15,0%)	16 (16,8%)	0,791
Demência	32 (12,0%)	16 (16,8%)	0,306
Epilepsia/convulsão	12 (4,5%)	1 (1,1%)	0,197
DRC	19 (7,1%)	11 (11,6%)	0,255
Depressão	22 (8,2%)	5 (5,3%)	0,471
DPOC	12 (4,5%)	3 (3,2%)	0,768
Alcoolismo	5 (1,9%)	5 (5,3%)	**0,136**
Esquizofrenia	4 (1,5%)	0 (0,0%)	0,577
Desnutrição	2 (0,7%)	2 (2,1%)	0,282
Doença hepática crônica	0 (0,0%)	2 (2,1%)	**0,068**
Medicações, n (%)			

Variáveis	Morte		Valor p
	Não (n = 267)	Sim (n = 95)	
BRA / IECA	139 (52,1%)	26 (27,4%)	**<0,001**
Diuréticos	98 (36,7%)	24 (25,3%)	**0,057**
Antidepressivos	39 (14,6%)	9 (9,5%)	0,223
Anticonvulsivantes	31 (11,6%)	8 (8,4%)	0,504
Opioides	15 (5,6%)	8 (8,5%)	0,458
Antagonista da aldosterona	14 (5,2%)	9 (9,5%)	0,228
AINE	10 (3,7%)	4 (4,2%)	0,766
Ciclofosfamida	1 (0,4%)	1 (1,1%)	0,457
UTI, n (%)	212 (79,4%)	84 (88,4%)	**0,072**
Tempo de internação, mediana [IIQ]	10 [6 – 15]	11 [7 – 20]	**0,086**
Tratamento hospitalar, n (%)			
NaCl 3%	61 (22,8%)	22 (23,2%)	1,000
Outra solução salina hipertônica	44 (16,5%)	14 (14,7%)	0,814
NaCl 0,9%	242 (90,6%)	85 (89,5%)	0,899
Solução glicosada a 5%	42 (15,7%)	12 (12,6%)	0,575
Furosemida	89 (33,3%)	47 (49,5%)	**0,008**
Restrição hídrica	62 (23,2%)	7 (7,4%)	**0,001**
Dieta rica em solutos	11 (4,1%)	4 (4,2%)	1,000
KCl	82 (30,7%)	25 (26,3%)	0,499
Volume de NaCl 0,9% no PS, n (%)			
• 0 ml	64 (24,0%)	27 (28,4%)	0,751
• até 499 ml	8 (3,0%)	4(4,2%)	
• 500 - 1000 ml	184 (68,9%)	60 (63,2%)	
• > 1000 ml	11 (4,1%)	4 (4,2%)	
Investigação etiológica, n (%)			
Cortisol < 5 mcg/dL (n = 81)	2 (2,9%)	0 (0,0%)	1,000
TSH > 10 (n = 159)	18 (14,0%)	6 (20,0%)	0,403
T4 livre < 0,7 ng/dL (n = 157)	5 (3,9%)	1 (3,6%)	1,000
Ureia > 40 mg/dL (n = 362)	89 (33,3%)	54 (56,8%)	**< 0,001**
Creatinina > 1,2 mg/dL (n = 362)	42 (15,7%)	31 (32,6%)	**< 0,001**
Potássio < 3,5 mmol/L (n = 362)	45 (16,9%)	9 (9,5%)	**0,117**
Ácido úrico baixo (n = 32)	17 (68,0%)	2 (28,6%)	**0,091**
Sódio urinário < 20 (n = 39)	5 (15,6%)	0 (0,0%)	0,563
Sódio urinário < 40 (n = 39)	12 (37,5%)	0 (0,0%)	**0,077**

Abreviaturas - DRC = doença renal crônica; DPOC = doença pulmonar obstrutiva crônica; BRA = bloqueador do receptor de angiotensina; IECA = inibidor da enzima conversora de angiotensina; AINE = anti-inflamatório não esteroide; UTI = unidade de terapia intensiva; IIQ = intervalo interquartil; NaCl = cloreto de sódio; KCl = cloreto de potássio; PS = pronto-socorro; TSH = hormônio estimulante da tireoide.
Nota - Para a variável idade, n = 361. Para a variável volume de NaCl 0,9%, n = 356. As variáveis contínuas são expressas como média e desvio padrão.
