# Supplementary material for: Overcorrection of severe hyponatremia, osmotic demyelination syndrome, and mortality: insights from two Brazilian centers
Source: J Bras Nefrol. 2026 Jan 23;48(1):e20250161. doi: 10.1590/2175-8239-JBN-2025-0161en (PMC12854713; doi:10.1590/2175-8239-JBN-2025-0161en)
Supplement: Translation

# Translation: Hipercorreção da hiponatremia grave, síndrome de desmielinização osmótica e mortalidade: percepções de dois centros brasileiros

**DOI:** 10.1590/2175-8239-JBN-2025-0161pt

**Authors:** Lívia de Azevêdo Cerqueira Reis, Maria Gabriela Motta Guimarães, Ananda Pires Bastos, Dyonatas Rodrigues da Mata, Daniel Henrique Lins e Silva, Paulo Novis Rocha

**Affiliations:** 1 Universidade Federal da Bahia, Programa de Pós-Graduação em Ciências da Saúde, Salvador, BA, Brasil.; 2 Universidade Federal da Bahia, Faculdade de Medicina da Bahia, Salvador, BA, Brasil.; 3 Universidade Federal da Bahia, Faculdade de Medicina da Bahia, Departamento de Medicina Interna e Apoio Diagnóstico, Salvador, BA, Brasil.

**Keywords:** Hiponatremia, Mielinólise Central da Ponte, Solução Salina, Síndrome de Secreção Inadequada de HAD

## Abstract

**Introdução:**

Hiponatremia grave (sódio ≤120 mmol/L) representa riscos clínicos significativos, incluindo encefalopatia e convulsões. A correção rápida inadvertida pode causar síndrome de desmielinização osmótica (SDO). Diretrizes atuais recomendam limitar correção de sódio a ≤8 mmol/L por 24 horas para minimizar o risco de SDO. Entretanto, estudos recentes sugerem que a hipercorreção pode não contribuir diretamente para mortalidade estando, inclusive, associada a melhores desfechos.

**Métodos:**

Estudo de coorte retrospectivo incluindo 362 pacientes com hiponatremia grave internados em dois hospitais terciários brasileiros. Definiu-se hipercorreção como aumento do sódio sérico >8 mmol/L em 24 horas ou >18 mmol/L em 48 horas. Utilizou-se regressão logística multivariada e análises ponderadas por escore de propensão para identificar preditores e desfechos associados à hipercorreção.

**Resultados:**

Hipercorreção ocorreu em 38,7% dos pacientes, enquanto SDO em apenas um (0,28%). Preditores independentes de hipercorreção incluíram idade mais jovem, menores níveis de sódio na admissão e volumes maiores de NaCl 0,9% administrados nos serviços de emergência; diagnóstico de câncer e uso de furosemida constituíram fatores protetores. A hipercorreção foi associada a menor mortalidade hospitalar e internações mais curtas, mesmo em análises multivariadas ponderadas por escore de propensão. Contudo, uma revisão detalhada dos casos de mortalidade não revelou relação causal direta entre taxa de correção de sódio e óbito.

**Conclusão:**

A hipercorreção da hiponatremia grave foi comum e associada a melhores desfechos clínicos, sem aumento significativo do risco de SDO. Entretanto, dada a natureza observacional dessa associação, são necessários ensaios clínicos randomizados antes que diretrizes atuais para taxa de correção possam ser reconsideradas.

## Introdução

Quando a ingestão de água excede a excreção renal, os pacientes desenvolvem hiponatremia. Como a concentração sérica de sódio determina a tonicidade do fluido extracelular (FEC), a hiponatremia cria um estado hipotônico que leva ao movimento da água do FEC para o fluido intracelular (FIC). A hiponatremia grave, definida por uma concentração sérica de sódio < 120 mmol/L, é particularmente perigosa e pode resultar em encefalopatia, coma, convulsões e óbito. Como mecanismo de proteção, os neurônios se adaptam por meio da redução do seu conteúdo de eletrólitos e osmólitos, a fim de minimizar o influxo de água e, assim, estabilizar o volume celular. No entanto, essas adaptações requerem tempo tanto para se desenvolverem quanto para serem revertidas. Quando a hiponatremia crônica é corrigida de forma muito rápida, o FEC relativamente hipertônico pode retirar água dos neurônios, ocasionando uma rápida redução no volume neuronal. Em pacientes suscetíveis, isso pode levar à síndrome de desmielinização osmótica (SDO)^1^.

O primeiro relato de caso de SDO foi publicado em 1959 por Adams et al.^2^ e, desde então, modelos animais confirmaram o papel da correção rápida da hiponatremia e sua patogênese^3,4^. Uma revisão sistemática recente demonstrou que a mediana do sódio sérico na admissão em 96 casos confirmados de SDO foi de 105 mmol/L, com mais de 90% dos casos apresentando níveis séricos de sódio < 120 mmol/L com taxas de correção > 10 mmol/L/dia^5^. Dados os riscos representados tanto pela hiponatremia grave em si quanto pela SDO relacionada ao tratamento, o manejo da hiponatremia tem sido objeto de debate de longa data^6,7^.

No passado, a controvérsia quanto à taxa de correção da hiponatremia grave levou a divergências significativas entre os especialistas. Alguns enfatizaram os riscos da encefalopatia hiponatrêmica e defenderam uma correção mais rápida^8–10^, enquanto outros expressaram maior preocupação com a SDO e recomendaram cautela na correção da hiponatremia grave^11–13^. Com o tempo, prevaleceu uma abordagem mais conservadora. A taxa recomendada de correção reduziu-se gradualmente de 1 mmol/L/hora para < 0,5 mmol/L/hora. As diretrizes norte-americanas de 2013 propuseram uma meta de 6 mmol/L/dia para pacientes com risco significativo de SDO, com limite de 8 mmol/L/dia^14^.

Limitar a correção da hiponatremia grave a < 8 mmol/L/dia pode ser desafiador, uma vez que os níveis séricos de sódio podem aumentar de forma não intencional em pacientes com causas reversíveis de hiponatremia. Por exemplo, na hiponatremia hipovolêmica, a reposição de volume suprime a secreção do hormônio antidiurético (ADH) mediada por barorreceptores, desencadeando aquarese e um aumento rápido e não intencional do sódio sérico^15^. Um amplo estudo multicêntrico norte-americano indicou que a hipercorreção ocorre em aproximadamente 40% dos pacientes, com fatores de risco que incluem idade mais jovem, sexo feminino, esquizofrenia, hiponatremia grave e baixo nível inicial de sódio na urina^16^. A hipercorreção da hiponatremia grave é considerada uma emergência médica^17^ e estratégias de manejo, como descontinuação da administração de cloreto de sódio e rediluição do sódio sérico com dextrose a 5% em água (D5W)^14^ e, se necessário, administração de desmopressina (DDAVP), são descritas em diretrizes recentes. Alguns especialistas chegaram a sugerir o uso profilático de DDAVP em casos selecionados, visando prevenir a aquarese e a hipercorreção^18^.

Estudos na América Latina sobre a frequência da hipercorreção da hiponatremia são escassos, e diferenças nos padrões de prática e no conhecimento das diretrizes para hiponatremia podem levar a variações na incidência de hipercorreção entre as regiões. Portanto, o objetivo inicial do nosso estudo foi avaliar a incidência e os fatores de risco para a hipercorreção da hiponatremia grave em dois hospitais terciários no Brasil. No entanto, publicações recentes na revista *NEJM Evidence* indicaram que, embora a hipercorreção da hiponatremia seja comum, a SDO é extremamente rara^19,20^. Além disso, pacientes com hiponatremia grave que apresentaram correção do sódio sérico > 10 mmol/L/dia—taxas consideradas hipercorreção pelos padrões atuais—apresentaram menor mortalidade intra-hospitalar e em 30 dias do que aqueles corrigidos de forma mais lenta, em conformidade com as diretrizes atuais^20^. Esses achados reacenderam o debate, com alguns autores sugerindo que as diretrizes atuais podem ser excessivamente conservadoras, potencialmente expondo os pacientes a um maior risco de mortalidade devido à hipocorreção^21,22^, enquanto outros afirmam que são necessárias mais evidências provenientes de ensaios clínicos randomizados antes que as diretrizes sejam modificadas^23^.

Considerando esses achados recentes, nosso estudo também teve como objetivo contribuir para o debate em curso, comparando a mortalidade hospitalar ajustada entre pacientes que sofreram hipercorreção e aqueles que seguiram as diretrizes. Além disso, buscamos determinar a causa imediata do óbito para avaliar se a hiponatremia ou sua taxa de correção desempenharam papel causal.

## Métodos

Desenho do Estudo e Seleção da Amostra: Este foi um estudo de coorte retrospectivo que analisou os prontuários eletrônicos de pacientes de duas instituições terciárias de saúde, uma filantrópica e outra privada, ambas localizadas em Salvador, Bahia, Brasil. Inicialmente, solicitamos aos departamentos de tecnologia da informação (TI) dessas instituições uma lista de todos os pacientes que apresentaram hiponatremia grave, definida como um valor inicial de sódio sérico < 120 mmol/L. O período do estudo foi de janeiro de 2016 a julho de 2019. Foram excluídos pacientes com idade inferior a 18 anos, aqueles que foram atendidos no pronto-socorro (PS) e depois receberam alta, aqueles com < 3 medições de sódio sérico durante a internação e aqueles cuja segunda medição de sódio sérico foi coletada ≥ 30 horas após a medição inicial.

Definição de Hipercorreção da Hiponatremia: A hipercorreção da hiponatremia foi definida como um aumento no nível sérico de sódio > 8 mmol/L/24 horas ou > 18 mmol/L/48 horas após a admissão. Para permitir a comparação com outros estudos, a frequência da hipercorreção também foi avaliada utilizando o critério de um aumento no nível sérico de sódio > 10 mmol/L/24 horas, mantendo o mesmo critério de > 18 mmol/L/48 horas.

Definição de Sódio Sérico na Admissão, Sódio Sérico em 24 horas e Sódio Sérico em 48 horas: O sódio sérico na admissão foi definido como a primeira medição de sódio sérico obtida na admissão hospitalar, geralmente no PS. Por se tratar de um estudo observacional, os níveis séricos de sódio nem sempre foram medidos exatamente 24 ou 48 horas após a admissão. Considerou-se aceitável um desvio de até 4 horas antes ou depois desses pontos temporais. Se não houvesse medição do sódio sérico disponível dentro dessa janela temporal, os valores de 24 e 48 horas eram estimados com base na taxa de variação do sódio sérico (mmol/L/h), utilizando-se os valores disponíveis. Caso um paciente apresentasse um aumento rápido do sódio sérico (> 8 mmol/L) durante as primeiras 24 horas, mas houvesse um declínio subsequente de tal forma que a variação em 24 horas fosse < 8 mmol/L, o caso ainda seria classificado como hipercorreção. Nesses casos, entretanto, foram realizadas intervenções específicas (por exemplo, suspensão da solução salina hipertônica ou administração de água livre/fluidos hipotônicos) com o objetivo de reduzir o sódio sérico.

Cálculo do Tamanho da Amostra: Em virtude da escassez de estudos que avaliem a frequência de hipercorreção da hiponatremia grave em nossa região, realizamos um estudo piloto em uma das instituições. A partir de uma lista de casos de hiponatremia grave fornecida pelo departamento de TI de uma determinada instituição, analisamos uma amostra aleatória de 50 pacientes e constatamos que a frequência de hipercorreção foi de 32%. Esse percentual foi então utilizado para o cálculo do tamanho amostral, realizado por meio da ferramenta online gratuita *Open Epi* (https://www.openepi.com/SampleSize/SSPropor.htm). Assumindo uma população infinita, uma frequência de hipercorreção de 32% e uma margem de erro de 5%, seria necessário analisar um mínimo de 335 pacientes. Antecipando que alguns dos pacientes triados seriam excluídos, os prontuários de aproximadamente 485 pacientes foram selecionados para revisão.

Aspectos Éticos: O projeto de pesquisa foi submetido e aprovado pelo Comitê de Ética em Pesquisa (CEP) de uma das instituições, sob o número de protocolo 3.474.662, e obtivemos uma declaração de concordância da outra instituição colaboradora. Por se tratar de um estudo retrospectivo envolvendo a análise de prontuários médicos, o CEP dispensou o termo de consentimento livre e esclarecido.

Coleta de Dados: O formulário de relato de caso incluiu dados demográficos, data de admissão, data de alta hospitalar ou óbito, disposição hospitalar (unidade de terapia intensiva ou enfermaria), se houve avaliação com nefrologista para hiponatremia, bem como informações sobre condições pré-existentes, tais como hipertensão arterial sistêmica, diabetes mellitus (DM), câncer, doença hepática crônica, doença renal crônica (DRC), epilepsia ou convulsões, acidente vascular cerebral, insuficiência cardíaca congestiva (ICC), doença pulmonar obstrutiva crônica (DPOC), além de depressão, transtorno afetivo bipolar, esquizofrenia, demência, desnutrição e histórico de alcoolismo. Foram listados os medicamentos de uso regular, desde que pertencentes às seguintes classes: diuréticos, inibidores da enzima conversora de angiotensina (IECA) ou bloqueadores do receptor de angiotensina (BRA), antidepressivos, anti-inflamatórios não esteroides (AINEs), anticonvulsivantes, opioides, antagonistas da aldosterona e ciclofosfamida. Para a investigação etiológica da hiponatremia, foram analisados laudos de tomografias computadorizadas (TC) de tórax e cabeça e ressonâncias magnéticas (RM) do crânio. Como parte da investigação etiológica, foram coletados os níveis de hormônio tireoidiano e cortisol sérico, se avaliados em qualquer momento durante a internação do paciente. Todas as medições de sódio sérico realizadas nas primeiras 48 horas foram avaliadas, incluindo as datas e horários de cada aferição. Em alguns casos, as medições de sódio sérico das 24 horas subsequentes (tempo total de coleta de 72 horas) também foram revisadas para estimar o nível de sódio em 48 horas. Todas as prescrições médicas emitidas durante as primeiras 48 horas após a admissão foram revisadas, em busca de prescrições de NaCl 0,9%, solução salina hipertônica (a 3% ou outra concentração > 0,9%) e soluções de dextrose. Além disso, também foram analisadas a administração de potássio, a existência de prescrições de restrição hídrica, a modificação da dieta para uma dieta hiperproteica e a administração de furosemida. Também foram registrados a primeira solução infundida no PS e o volume aplicado; no entanto, não foi possível confirmar se todo o volume prescrito (ou apenas parte dele) foi administrado. A coleta de dados foi realizada pelo pesquisador principal (PP) e por três estudantes de medicina (APB, DRM e DHLS) treinados pelo PP. Os dados referentes à causa do óbito foram coletados por dois nefrologistas assistentes (o PP e MGMG). Todos os dados coletados foram inseridos exclusivamente pelo PP em um banco de dados do Microsoft Excel e, em seguida, transferidos para o SPSS para análise estatística.

Análise Estatística: As variáveis contínuas com distribuição normal foram resumidas utilizando média e desvio padrão, enquanto as variáveis com distribuição não normal foram resumidas por meio de mediana e intervalo interquartil. As variáveis categóricas foram resumidas com frequências absolutas e relativas. Os pacientes foram estratificados de acordo com a presença ou ausência de hipercorreção da hiponatremia. A comparação das variáveis quantitativas entre os dois grupos foi realizada utilizando o teste t de Student, e as variáveis categóricas foram comparadas por meio do teste qui-quadrado. Para comparar a média de sódio sérico na admissão e em 24 horas dentro do mesmo grupo, utilizamos o teste t pareado. Para avaliar se houve uma tendência entre o volume de NaCl 0,9% administrado no PS e a magnitude do aumento do sódio sérico em 24 horas, utilizamos o teste de tendência de Jonckheere-Terpstra. A regressão logística múltipla foi empregada para identificar preditores independentes de hipercorreção da hiponatremia e mortalidade. Além disso, calculamos ORs ajustados por escore de propensão para mortalidade. Os preditores independentes de hipercorreção da hiponatremia foram utilizados para criar escores de propensão. Os pacientes do grupo com hipercorreção receberam pesos iguais ao inverso de seus escores de propensão, enquanto os demais pacientes receberam pesos iguais ao inverso de (1 – escore de propensão). Por fim, utilizamos o conjunto de dados ponderados para realizar uma regressão logística tendo a mortalidade como desfecho e a hipercorreção da hiponatremia como preditor. Todas as análises foram realizadas com o software SPSS para Windows versão 20. Um valor de p < 0,05 nas análises finais foi considerado estatisticamente significativo.

## Resultados

### Descrição da Amostra (N = 362) e Frequência de Hipercorreção da Hiponatremia

A estratégia de amostragem é apresentada na Figura 1. A amostra final consistiu em 362 pacientes adultos internados com hiponatremia grave. A idade média foi de 75,6 ± 13,6 anos, e 65,5% eram mulheres. Hipertensão (82,3%) e DM (76,2%) foram as comorbidades mais prevalentes e a maioria dos pacientes (81,8%) foi admitida na unidade de terapia intensiva (UTI), destacando a gravidade clínica dos casos. A mortalidade geral foi de 26,2%.

**Figura 1.** Fluxograma descrevendo o processo de amostragem.

Notas – Inicialmente, todos os pacientes com registros de Na sérico < 120 mmol/L na admissão foram selecionados a partir dos prontuários eletrônicos de dois hospitais terciários, no período de janeiro de 2016 a julho de 2019. Após a exclusão de registros duplicados, permaneceram 1.236 pacientes. Posteriormente, realizamos uma amostragem aleatória e reduzimos o tamanho da amostra para 484 pacientes, um número bem superior ao mínimo calculado de 335 pacientes necessários para a amostra final. Em seguida, revisamos os prontuários e aplicamos os critérios de exclusão: 47 pacientes foram excluídos devido ao fato de que o “Na1” (primeira medição de sódio sérico após a admissão; o sódio na admissão foi referido como “Na0”) foi coletado > 48 horas após a admissão, ou de que o “Na2” (segunda medição de sódio sérico após a admissão) foi coletado > 30 horas após o “Na1”. Adicionalmente, 46 pacientes foram excluídos por apresentarem menos de três medições de sódio sérico durante a hospitalização. Vinte e um pacientes foram excluídos por não terem sido hospitalizados e oito por terem idade inferior a 18 anos. A amostra final de 362 pacientes foi composta por pacientes adultos internados com hiponatremia grave, com três ou mais medições de sódio sérico durante a internação, em um intervalo que permitiu a detecção de hipercorreção.

À chegada ao PS, o sódio sérico médio na admissão foi de 113,3 ± 4,9 mmol/L, variando entre 94 e 119 mmol/L (Figura S1). A maioria dos pacientes (73,2%) recebeu NaCl 0,9% no PS, com um volume médio de 494,7 ± 392,8 mL. Apenas 6 pacientes receberam NaCl 3% ou outra solução hipertônica no PS. Houve uma correlação estatisticamente significativa entre o volume de NaCl 0,9% administrado no PS e a variação do sódio sérico em 24 horas (p para tendência < 0,001) (Figura 2). Nas primeiras 24 horas, a variação média do sódio sérico foi um aumento de 6,65 ± 5,31 mmol/L, variando de uma redução de 6 mmol/L a um aumento de 24 mmol/L. Com base na variação do sódio sérico nas primeiras 24 horas, estratificamos a evolução da natremia em seis categorias: redução do sódio sérico em 7,2%; sem alteração em 3,9%; aumento insuficiente do sódio sérico (1,0 a 3,9 mmol/L) em 18,1%; aumento ideal do sódio sérico (4,0 a 8,0 mmol/L) em 31,6%; hipercorreção em 35,5% (aumento > 8 mmol/L em 24 horas); e hipercorreção com rediluição bem-sucedida, de modo que a diferença entre o sódio ao final de 24 horas e o sódio na admissão foi < 8 mmol/L em 3,3% (Figura S2).

**Figura 2.** Variação do sódio sérico em 24 horas e volume de NaCl 0,9% administrado no PS.

A incidência de hipercorreção considerando os critérios de 24 horas (> 8 mmol/L) e 48 horas (> 18 mmol/L) foi de 38,7% (Tabela S1). A incidência de hipercorreção diminuiu para 23% quando o critério de 24 horas foi alterado de > 8 para > 10 mmol/L, enquanto o critério de 48 horas permaneceu constante (> 18 mmol/L). Isso foi feito exclusivamente para comparar nossos dados com os de outros estudos. Todas as análises subse­quentes foram realizadas considerando o critério de 24 horas > 8 mmol/L.

Houve apenas um caso de SDO (0,28%).

### Descrição da Amostra Estratificada por Hipercorreção da Hiponatremia

A Tabela 1 apresenta os dados demográficos, clínicos e de desfecho dos pacientes, estratificados pela presença ou ausência de hipercorreção da hiponatremia. Os achados notáveis no grupo com hipercorreção incluíram idade mais jovem, sódio sérico mais baixo na admissão, menos casos de câncer, menor uso de furosemida, menor mortalidade e tempo de permanência hospitalar mais curto; os pacientes com hipercorreção utilizaram maior quantidade de NaCl 0,9% e D5W, apresentaram mais casos de hipocalemia e utilizaram mais cloreto de potássio (KCl).

**Tabela 1:** Dados demográficos, clínicos e de desfecho de 362 pacientes internados com hiponatremia grave, estratificados de acordo com a presença ou ausência de hipercorreção da hiponatremia

Variáveis	Hipercorreção		Valor de p
	Não (n = 222)	Sim (n = 140)	
Idade	76,81 ± 13,27	73,57 ± 13,85	**0,027**
Sexo feminino	144 (64,9%)	93 (66,4%)	0,848
Sódio sérico na admissão (mmol/L)	114,23 ± 4,37	111,85 ± 5,40	**<0,001**
Comorbidades, n (%)			
Hipertensão	180 (81,1%)	118 (84,3%)	0,524
Diabetes	78 (35,1%)	48 (34,3%)	0,959
Câncer	56 (25,2%)	22 (15,7%)	**0,044**
Acidente vascular cerebral	34 (15,3%)	31 (22,1%)	0,132
Insuficiência cardíaca	37 (16,7%)	19 (13,6%)	0,520
Demência	28 (12,6%)	20 (14,3%)	0,766
Epilepsia/convulsão	15 (6,8%)	16 (11,4%)	0,176
DRC	18 (8,1%)	12 (8,6%)	1,000
Depressão	15 (6,8%)	12 (8,6%)	0,664
DPOC	12 (5,4%)	3 (2,1%)	0,213
Alcoolismo	8 (3,6%)	2 (1,4%)	0,368
Esquizofrenia	3 (1,4%)	1 (0,7%)	0,961
Desnutrição	3 (1,4%)	1 (0,7%)	0,961
Doença hepática crônica	2 (0,9%)	0 (0,0%)	0,690
Medicamentos, n (%)			
BRA/Inibidor da ECA	95 (42,8%)	70 (50,0%)	0,218
Diuréticos	69 (31,1%)	53 (37,9%)	0,225
Antidepressivo	33 (14,9%)	15 (10,7%)	0,330
Anticonvulsivante	22 (9,9%)	17 (12,1%)	0,622
Opioide	17 (7,7%)	6 (4,3%)	0,285
Antagonista da aldosterona	16 (7,2%)	7 (5,0%)	0,537
AINE	11 (5,0%)	3 (2,1%)	0,284
Ciclofosfamida	2 (0,9%)	0 (0,0%)	0,690
UTI, n (%)	180 (81,1%)	116 (82,9%)	0,775
Óbito, n (%)	72 (32,4%)	23 (16,4%)	**0,001**
SDO, n (%)	0 (0,0%)	1 (0,7%)	0,387
Tempo de permanência, mediana [IIQ]	11 [7–16,5]	9 [6–14]	**0,024**
Tratamento hospitalar, n (%)			
Solução salina hipertônica a 3%	49 (22,0%)	34 (24,2%)	0,719
Outras soluções salinas hipertônicas	37 (16,6%)	21 (15,0%)	0,784
NaCl 0,9%	193 (86,9%)	134 (95,7%)	**0,010**
Solução de dextrose a 5%	24 (10,8%)	30 (21,4%)	**0,009**
Furosemida	98 (44,1%)	38 (27,1%)	**0,002**
Restrição hídrica	49 (22,0%)	19 (13,5%)	0,091
Dieta rica em solutos	11 (4,8%)	4 (2,8%)	0,481
KCl	56 (25,2%)	51 (36,4%)	**0,031**
Volume de NaCl 0,9% no PS, n (%)			
0 ml	72 (33,2%)	19 (13,7%)	**<0,001**
até 499 ml	6 (2,8%)	6 (4,3%)	
500–1000 ml	136 (62,7%)	108 (77,7%)	
> 1000 ml	3 (1,4%)	6 (4,3%)	
Investigação etiológica, n (%)			
Cortisol < 5 mcg/dL (n = 81)	2 (3,5%)	0 (0,0%)	0,885
TSH > 10 (n = 159)	15 (15,3%)	9 (14,8%)	1,000
T4 livre < 0,7 ng/dL (n = 157)	4 (4,2%)	2 (3,3%)	1,000
Ureia > 40 mg/dL (n = 362)	91 (41%)	52 (37,1%)	0,536
Creatinina > 1,2 mg/dL (n = 362)	45 (20,3%)	28 (20,0%)	1,000
Potássio < 3,5 mmol/L (n = 362)	20 (9,0%)	34 (24,3%)	**<0,001**
Baixo nível de ácido úrico (n = 32)	9 (52,9%)	10 (66,7%)	0,668
Sódio na urina < 20 (n = 39)	3 (10,7%)	2 (18,2%)	0,924
Sódio na urina < 40 (n = 39)	7 (25,0%)	5 (45,5%)	0,390

Abreviaturas – Para a variável idade, n = 361. Para a variável volume de NaCl 0,9%, n = 356. Variáveis contínuas são expressas como média e desvio padrão. DRC: doença renal crônica; DPOC: doença pulmonar obstrutiva crônica; BRA: bloqueador do receptor da angiotensina; ECA: enzima conversora da angiotensina; AINE: anti-inflamatório não esteroide; UTI: unidade de terapia intensiva; SDO: síndrome de desmielinização osmótica; IIQ: intervalo interquartil; NaCl: cloreto de sódio; KCl: cloreto de potássio; PS: pronto-socorro; TSH: hormônio estimulante da tireoide.

A Figura 3a apresenta os resultados do sódio sérico. Primeiro, a média do sódio sérico na admissão foi significativamente menor no grupo com hipercorreção (111,9 mmol/L em comparação a 114,2 mmol/L; p < 0,001). Em segundo lugar, os níveis médios de sódio sérico em 24 e 48 horas foram significativamente mais elevados no grupo com hipercorreção (24 horas: 123,6 em comparação a 117,6 mmol/L, p < 0,001; 48 horas: 128,2 em comparação a 121,9 mmol/L, p < 0,001). É importante notar que o nível médio de sódio em 24 horas no grupo com hipercorreção é numericamente mais elevado do que o nível médio de sódio em 48 horas no grupo sem hipercorreção. A Figura 3b indica que o aumento médio do sódio sérico em 24 horas no grupo com hipercorreção foi de 11,8 mmol/L, em comparação a apenas 3,4 mmol/L no grupo sem hipercorreção (p < 0,001); o aumento médio do sódio sérico em 48 horas também foi significativamente maior no grupo com hipercorreção (16,5 mmol/L em comparação a 7,8 mmol/L, p < 0,001).

**Figura 3.** Evolução do sódio sérico nas primeiras 48 horas. (a) Sódio sérico na admissão, em 24 horas e em 48 horas estratificado por hipercorreção da hiponatremia; (b) variação do sódio sérico em 24 horas e em 48 horas estratificado por hipercorreção da hiponatremia.

A Figura 4 demonstra que o grupo com hipercorreção recebeu, em média, um volume signi­ficativamente maior de NaCl 0,9% no PS em com­paração àqueles que não realizaram hipercorreção (623,02 ± 535,39 vs. 433,18 ± 387,34 ml; p < 0,001).

**Figura 4.** Volume de NaCl 0,9% administrado no pronto-socorro estratificado por hipercorreção da hiponatremia.

### Preditores Independentes de Hipercorreção da Hiponatremia

Para identificar preditores independentes de hipercorreção da hiponatremia, utilizamos regressão logística múltipla. O modelo incluiu as variáveis associadas ao desfecho nas análises univariadas. A Tabela 2 apresenta os resultados de dois modelos. No primeiro modelo, as variáveis contínuas idade, sódio sérico na admissão e volume de NaCl 0,9% no PS foram estratificadas utilizando o seu valor médio como ponto de corte. No segundo modelo, estas três variáveis foram avaliadas em seu formato contínuo.

**Tabela 2:** Preditores independentes de hipercorreção da hiponatremia

Variáveis independentes	OR	IC 95%	Valor de p
Modelo 1			
NaCl 0,9% ≥ 500 ml no PS	2,10	1,22–3,61	0,007
Sódio sérico na admissão ≤ 113 mmol/L	2,40	1,52–3,80	<0,001
Idade ≤ 75 anos	1,96	1,22–3,13	0,005
Câncer	0,45	0,25–0,82	0,008
Uso de furosemida	0,52	0,32–0,84	0,008
Modelo 2			
NaCl 0,9% (cada 100 ml) no PS	1,10	1,04–1,17	0,002
Sódio sérico na admissão	0,91	0,87–0,95	<0,001
Idade	0,98	0,97–0,99	0,035
Câncer	0,55	0,31–0,99	0,047
Uso de furosemida	0,46	0,28–0,76	0,002

Abreviaturas – NaCl: cloreto de sódio.

Notas – No Modelo 1, as variáveis contínuas foram categorizadas utilizando a média como ponto de corte. No Modelo 2, as variáveis contínuas foram tratadas como tal. Para que a variável relacionada ao volume de NaCl administrado no PS tivesse maior relevância clínica, multiplicamos os betas por 100 e, em seguida, calculamos o logaritmo natural, de modo que os *odds ratios* expressassem o risco para cada 100 ml de solução.

A análise multivariada revelou que idade mais jovem, sódio sérico mais baixo na admissão e maior volume de NaCl 0,9% administrado no PS estiveram associados a maiores chances de hipercorreção. Em contrapartida, o diagnóstico de câncer e o uso de furosemida estiveram associados a menores chances de hipercorreção.

### Impacto da Hipercorreção da Hiponatremia

A mortalidade intra-hospitalar não ajustada foi significativamente menor em pacientes que apresentaram hipercorreção da hiponatremia (16,4% versus 32,4%; p = 0,001) (Figura 5). Os pacientes que foram a óbito apresentaram níveis séricos de sódio significativamente mais baixos na admissão (112,30 ± 5,8 mmol/L vs. 113,67 ± 4,5 mmol/L; p = 0,039), em 24 horas (117,58 ± 5,7 mmol/L vs. 120,77 ± 6,2 mmol/L; p < 0,001) e em 48 horas (122,04 ± 6,9 mmol/L vs. 125,16 ± 6,6 mmol/L; p < 0,001) (Figura S3). Isso se traduziu em um aumento significativamente menor do sódio sérico naqueles que foram a óbito em comparação aos sobreviventes, tanto em 24 horas (5,27 ± 4,5 mmol/L vs. 7,1 ± 5,5 mmol/L; p = 0,002) quanto em 48 horas (9,47 ± 6,0 mmol/L vs 11,72 ± 6,6 mmol/L; p = 0,016) (Figura S3).

**Figura 5.** Mortalidade hospitalar estratificada por hipercorreção da hiponatremia.

A associação ajustada entre a hipercorreção da hiponatremia e a menor mortalidade foi avaliada por meio de análise multivariada. Para a seleção das variáveis incluídas no modelo multivariado, realizamos inicialmente análises univariadas estratificando a coorte por mortalidade (Tabela S2). As variáveis significativamente associadas ao aumento da mortalidade nas análises univariadas foram: idade mais avançada, sexo masculino, menor concentração sérica de sódio na admissão, diagnóstico de câncer, uso de furosemida durante a hospitalização e níveis mais elevados de ureia e creatinina na admissão. A hipercorreção da hiponatremia, o uso domiciliar de IECA/BRA e a restrição hídrica durante a hospitalização estiveram associados à redução da mortalidade. O alcoolismo, a doença hepática crônica, a admissão em UTI e o tempo de permanência hospitalar mais prolongado estiveram marginalmente associados ao aumento da mortalidade. O diagnóstico de hipertensão, o uso domiciliar de diuréticos, a hipocalemia, a hipouricemia e o baixo nível de sódio na urina foram marginalmente associados à redução da mortalidade. Como havia apenas dois pacientes com doença hepática crônica e muitos dados ausentes para ácido úrico e sódio na urina, essas variáveis foram excluídas de análises subsequentes. Todas as demais variáveis foram incluídas em uma regressão logística multivariada *stepwise backward*. Conforme demonstrado na Tabela 3, a hipercorreção da hiponatremia foi associada a uma redução de 64% nas chances de mortalidade. O uso domiciliar de IECA/BRA e a restrição hídrica no hospital também estiveram associados à redução da mortalidade. Idade mais avançada, sexo masculino, câncer, níveis séricos de sódio mais baixos na admissão, creatinina sérica mais elevada na admissão e uso de furosemida durante a internação estiveram associados a maiores chances de mortalidade intra-hospitalar. Por fim, utilizamos preditores basais de hipercorreção para criar escores de propensão e, em seguida, realizamos uma análise de regressão logística ponderada por escore de propensão, com a mortalidade como desfecho e a hipercorreção como preditor. Nessa análise, a hipercorreção da hiponatremia manteve-se associada a uma redução de 45% nas chances de mortalidade (OR 0,548; IC 95% 0,389 a 0,772; p < 0,001).

**Tabela 3:** Preditores independentes de óbito

Variáveis independentes	OR	IC 95%	Valor de p
Hipercorreção da hiponatremia	0,364	0,196–0,675	0,001
Idade	1,041	1,017–1,066	<0,001
Sexo masculino	2,088	1,181–3,691	0,011
BRA/Inibidor da ECA (domiciliar)	0,382	0,213–0,684	0,045
Câncer	2,370	1,268–4,430	0,007
Sódio sérico na admissão ≤ 113 mmol/L	2,166	1,218–3,851	0,008
Creatinina sérica na admissão > 1,2 mg/dL	2,810	1,482–5,329	0,002
Furosemida (hospital)	1,788	1,012–3,157	0,001
Restrição hídrica (hospital)	0,186	0,073–0,473	<0,001

Abreviaturas – BRA: bloqueador do receptor da angiotensina; ECA: enzima conversora da angiotensina.

Notas – Variáveis inseridas na etapa inicial: idade, sexo, hipertensão, câncer, alcoolismo, BRA/inibidor da ECA (domiciliar), diurético (domiciliar), furosemida (hospital), restrição hídrica (hospital), creatinina sérica na admissão > 1,2 mg/dL, ureia sérica na admissão > 40 mg/dL, hipercorreção da hiponatremia, potássio sérico na admissão < 3,5 mmol/L, sódio sérico na admissão ≤ 113 mmol/L, admissão em UTI, tempo de permanência hospitalar.

### Causa de Óbito

Para avaliar se a taxa de correção do sódio sérico teve influência direta na mortalidade, analisamos cuidadosamente os prontuários de todos os 95 pacientes que foram a óbito. Conforme mostrado na Tabela 4, a sepse foi a principal causa de óbito (55,8%). Condições sobrepostas foram frequentes; por exemplo, alguns pacientes com sepse também apresentaram câncer ou doença cardíaca, embora essas comorbidades não tenham sido consideradas a causa primária do óbito. De forma semelhante, o câncer (15,8%) foi atribuído como causa do óbito quando a progressão da doença foi identificada como o principal fator. A aspiração de conteúdo gástrico (13,7%) foi considerada como uma causa potencial de óbito por hiponatremia, identificada por meio da revisão ativa dos prontuários; outras comorbidades, como câncer ou infecção, podem ter estado presentes, mas foram secundárias. Os óbitos de causa cardíaca (7,4%) incluíram descompensação aguda de insuficiência cardíaca congestiva, choque cardiogênico e complicações decorrentes de cirurgia cardíaca. Outras causas incluíram acidente vascular cerebral (4,2%), hemorragia (2,1%) e SDO (1,1%). Todos os 13 óbitos por aspiração de conteúdo gástrico foram analisados detalhadamente; 11 (15,3%) ocorreram em pacientes que não apresentaram hipercorreção, enquanto somente 2 (8,7%) ocorreram no grupo com hipercorreção. Em 8 desses 13 óbitos, a aspiração de conteúdo gástrico não pôde ser associada à hiponatremia (devido ao início de cuidados paliativos, melhora significativa da hiponatremia ou outras causas de aspiração). Portanto, 5 óbitos foram considerados potencialmente associados à hiponatremia grave, 4 (5,6%) dos quais ocorreram no grupo sem hipercorreção e 1 (4,3%) ocorreu no grupo com hipercorreção. Constatamos um caso de SDO (1,1%), que ocorreu no grupo com hipercorreção (Tabela 4).

**Tabela 4:** Causas de óbito estratificadas por hipercorreção da hiponatremia

Causas do óbito	Total (n = 95)	Hipercorreção	
		Não (n = 72)	Sim (n = 23)
Sepse	53 (55,8%)	37 (51,4%)	16 (69,6%)
Câncer	15 (15,8%)	14 (19,4%)	1 (4,3%)
Aspiração de conteúdo gástrico	13 (13,7%)	11 (15,3%)	2 (8,7%)
Possivelmente relacionado à hiponatremia	5 (5,3%)	4 (5,6%)	1 (4,3%)
Pacientes em cuidados paliativos	3 (3,2%)	2 (2,8%)	1 (4,3%)
Provavelmente não relacionado à hiponatremia	5 (5,3%)	5 (6,9%)	0 (0,0%)
Cardíaca	7 (7,4%)	7 (9,7%)	0 (0,0%)
Acidente vascular cerebral	4 (4,2%)	2 (2,8%)	2 (8,7%)
Hemorragia	2 (2,1%)	1 (1,4%)	1 (4,3%)
SDO	1 (1,1%)	0 (0,0%)	1 (4,3%)

Abreviaturas – SDO: síndrome de desmielinização osmótica.

Notas – Dos 13 casos de aspiração de conteúdo gástrico, 3 estavam em cuidados paliativos e 5 não apresentavam hiponatremia grave ou tinham outras razões para encefalopatia no momento da aspiração de conteúdo gástrico. Portanto, somente 5 casos foram considerados possivelmente relacionados à hiponatremia grave. Destes, 4 não apresentaram hipercorreção e 1 apresentou hipercorreção da hiponatremia.

## Discussão

### Declaração Geral

A frequência de hipercorreção da hiponatremia grave em pacientes internados em dois hospitais terciários brasileiros foi de 38,7%. Maior gravidade da hiponatremia, maiores volumes de infusão de NaCl 0,9% no PS e menor idade aumentaram o risco de hipercorreção. O diagnóstico de câncer e o uso de furosemida estiveram associados a um menor risco de hipercorreção. Por fim, a hipercorreção da hiponatremia esteve associada a um tempo de permanência hospitalar mais curto e menores chances de mortalidade intra-hospitalar.

### Frequência de Hipercorreção

A frequência de hipercorreção da hiponatremia foi estudada por outros grupos. Um estudo multicêntrico realizado na Pensilvânia avaliou 1490 pacientes internados com hiponatremia grave e encontrou frequências de hipercorreção quase idênticas às do nosso estudo: 41% quando utilizado o critério > 8 mmol/L/24 horas e 26% com o critério > 10 mmol/L/24 horas^16^. Outro estudo, também envolvendo pacientes com hiponatremia grave, observou uma frequência de hipercorreção de 27,9% utilizando o critério > 10 mmol/L/24 horas^24^. Portanto, nossos resultados são consistentes com o que tem sido relatado na literatura e sugerem que a hipercorreção da hiponatremia é um fenômeno global, não limitado aos centros por nós estudados. Até onde temos conhecimento, este é o primeiro estudo sobre o tema na América Latina.

### Fatores de Risco para Hipercorreção

Mesmo dentro dessa amostra de pacientes com hiponatremia grave, quanto mais baixo o sódio sérico na admissão, maiores as chances de hipercorreção. O sódio sérico médio na admissão foi significativamente mais baixo no grupo com hipercorreção (111,9 ± 5,4 mmol/L versus 114,2 ± 4,4 mmol/L; p < 0,001), e um caso de sódio na admissão ≤ 113 mmol/L foi associado a uma probabilidade 2,4 vezes maior de hipercorreção (p < 0,001). Estudos anteriores também identificaram a gravidade da hiponatremia como um fator de risco para hipercorreção. Um estudo norte-americano publicado em 2007 avaliou 62 pacientes tratados com solução salina hipertônica e constatou que o nível médio de sódio sérico na admissão foi menor naqueles que apresentaram hipercorreção em comparação aos que não apresentaram (111,9 ± 1,5 mmol/L versus 118,5 ± 0,8 mmol/L; p = 0,002)^25^. Um estudo retrospectivo, de centro único, que analisou 56 pacientes com níveis de sódio < 125 mmol/L, constatou que o nível de sódio na admissão teve um impacto significativo na hipercorreção. Os autores observaram que cada aumento de 1 mmol/L no valor inicial de sódio reduziu o risco de hipercorreção em 16% (p = 0,037)^26^. Em nosso estudo, cada aumento de 1 mmol/L no sódio sérico na admissão reduziu as chances de hipercorreção em 9% (p < 0,001).

A terapia ideal para pacientes com hiponatremia grave permanece controversa. Alguns médicos ainda utilizam fórmulas para orientar o uso de infusões contínuas de soluções de NaCl. No entanto, devido à elevada frequência de hipercorreção ao aplicar tal estratégia, diversos especialistas, incluindo as diretrizes norte-americanas e europeias, recomendam o uso de pequenos bolus fixos (100–150 mL) de solução salina hipertônica para casos de hiponatremia grave e aguda^27^. Em nosso estudo, apenas 1,6% dos pacientes foram tratados com solução salina hipertônica no PS. Uma possível explicação seria a presença de sintomas leves que não justificassem o uso da solução hipertônica. Entretanto, não podemos descartar a possibilidade de que a limitada familiaridade com as soluções hipertônicas ou com as diretrizes atuais, bem como uma baixa percepção do risco de hipercorreção, possam ter influenciado a decisão terapêutica. A maioria de nossos pacientes foi tratada com NaCl 0,9% no PS (73,2%). Entre eles, 68,5% receberam volumes de NaCl 0,9% entre 500 e 1000 ml. A administração de volumes de NaCl 0,9% > 500 ml no PS, um tratamento comum para pacientes hipovolêmicos, duplicou o risco de hipercorreção. Para cada 100 ml adicionais de NaCl 0,9% infundidos no PS, o risco de hipercorreção aumentou em 10%.

Observamos uma relação inversa entre idade e hipercorreção. Para cada ano adicional de idade, as chances de hipercorreção foram reduzidas em 2%. A média de idade dos pacientes em nossa amostra foi de 75,6 ± 13,6 anos, mas o grupo com hipercorreção apresentou uma média de idade mais baixa (73,57 ± 13,85 vs. 76,81 ± 13,27 anos; p = 0,027). Os pacientes acima da idade média apresentaram quase o dobro de chances de hipercorreção. Essa associação inversa entre idade e hipercorreção também foi descrita por outros autores [4, 5, 8], mas a causa dessa associação não é clara.

Observamos dois fatores de proteção contra a hipercorreção: o câncer e o uso de furosemida. Pacientes com câncer apresentaram menor risco de hipercorreção, possivelmente porque aqueles com síndrome da antidiurese inapropriada (SIAD) estão protegidos da autocorreção devido aos níveis constantemente elevados de ADH. Também observamos que, embora a furosemida aumente a depuração renal de água, os pacientes que a utilizaram em nosso estudo apresentaram menores chances de hipercorreção. Nossa hipótese é que o uso da furosemida tenha atuado como um indicador para diagnósticos menos propensos à autocorreção, como SIAD ou hiponatremia hipervolêmica.

### Impacto da Hipercorreção

Pacientes que apresentaram hipercorreção da hiponatremia tiveram uma mediana de permanência hospitalar 2 dias menor do que aqueles que não apresentaram hipercorreção (9 versus 11 dias; p = 0,024). Isso está em consonância com o trabalho de Seethapathy et al.^20^, que demonstrou permanência hospitalar significativamente mais curta para pacientes que sofreram correção > 10 mmol/L/dia em comparação ao grupo que sofreu correção < 6 mmol/L/dia. Em concordância adicional com os dados de Seethapathy et al.^20^, também encontramos menor mortalidade intra-hospitalar em pacientes que apresentaram hipercorreção em comparação àqueles que não apresentaram (16,4% vs. 32,4%; p = 0,001). A associação entre hipercorreção da hiponatremia e menor mortalidade persistiu após o ajuste para covariáveis relevantes em análises multivariadas e em análises ponderadas por escore de propensão. Uma recente metanálise realizada por Ayus et al.^22^, que sintetizou dezesseis estudos de coorte envolvendo 11.811 pacientes com hiponatremia grave, indicou que a correção rápida esteve associada a menos óbitos hospitalares em comparação com uma correção mais lenta; além disso, a correção rápida não esteve associada a um aumento estatisticamente significativo do risco de SDO.

### Hiponatremia como Causa Direta de Óbito?

Embora a hiponatremia aguda e extrema possa causar morte direta por herniação cerebral se tratada de forma inadequada, essa não foi a razão pela qual os pacientes da nossa coorte foram a óbito. A possibilidade de que uma hiponatremia menos extrema, com curso mais crônico, possa contribuir para a mortalidade é controversa. Após uma revisão cuidadosa dos prontuários de todos os 95 pacientes que foram a óbito em nossa coorte, identificamos 5 (5,3%) casos de aspiração de conteúdo gástrico que poderiam ter sido precipitados por encefalopatia hiponatrêmica; 4 no grupo sem hipercorreção (5,6%) e 1 no grupo com hipercorreção (4,3%). Portanto, postulamos que a associação entre hipercorreção e menor mortalidade não é causal. Por exemplo, a associação relatada entre gravidade da hiponatremia e maior mortalidade em pacientes com insuficiência cardíaca e cirrose pode simplesmente indicar que a hiponatremia constitui um marcador do grau de desequilíbrio hemodinâmico causado pela doença subjacente. De modo semelhante, a associação entre hipercorreção e menor mortalidade em estudos observacionais pode não ser causal, mas sim decorrente de fatores de confusão. Em vez de demonstrar que uma correção mais rápida da hiponatremia protege os pacientes contra óbitos por hiponatremia, a hipercorreção pode estar simplesmente identificando um grupo de pacientes *com tendência a* uma correção mais rápida devido à natureza de sua doença subjacente. Uma das principais causas de hipercorreção da hiponatremia é o tratamento da hipovolemia, o que levanta a questão sobre a possibilidade de a hipercorreção de fato identificar pacientes com hipovolemia—que poderiam estar menos gravemente doentes e, portanto, menos propensos ao óbito. Por outro lado, a ausência de hipercorreção pode refletir pacientes com níveis constantemente elevados de ADH devido a SIAD induzida por câncer, cirrose ou insuficiência cardíaca. Embora tenhamos ajustado para essas variáveis em análises multivariadas, não se pode excluir a possibilidade de confusão residual.

### Limitações do Estudo

Este estudo apresenta diversas limitações, a maioria delas relacionadas à sua natureza observacional. Primeiro, foi necessário estimar os valores séricos de sódio em 24 e 48 horas em alguns pacientes, uma vez que nem todos possuíam medições de sódio nesses momentos específicos; uma estratégia semelhante foi recentemente utilizada por Seethapathy et al.^20^. Em segundo lugar, como não foi possível determinar o volume exato de solução salina infundida no PS, baseamo-nos nos volumes prescritos e verificados pela equipe de enfermagem, sem a certeza de que todo o volume prescrito tenha sido de fato infundido. O momento da utilização da furosemida também não foi registrado. Suspeitamos que o uso de furosemida tenha atuado como um indicador para casos de SIAD e hipervolemia, o que explicaria seu efeito protetor contra a hipercorreção, uma vez que, nessas condições clínicas, a hipercorreção é menos provável devido aos níveis persistentemente elevados de ADH. Em terceiro lugar, os dados sobre os sintomas dos pacientes, o estado volêmico preciso e o diagnóstico etiológico da hiponatremia também não estavam sistematicamente disponíveis nos registros, o que é uma limitação importante, já que essas variáveis orientam o manejo da hiponatremia na prática diária. Quarto, não dispúnhamos dos níveis séricos de glicose medidos concomitantemente com o sódio sérico na admissão, de modo a avaliar a hiponatremia hiperosmolar. Quinto, apesar do controle das covariáveis relevantes, a associação entre hipercorreção e menor mortalidade ainda poderia ser decorrente de fatores de confusão residuais. Após uma revisão detalhada dos registros médicos de todos os pacientes que foram a óbito, não foi possível estabelecer que a hiponatremia estivesse causalmente relacionada ao excesso de óbitos no grupo que apresentou correção mais lenta.

## Conclusões

A hipercorreção da hiponatremia grave foi comum nos dois centros brasileiros estudados, especialmente em pacientes mais jovens, com níveis séricos de sódio mais baixos na admissão e que receberam maior quantidade de solução salina no PS; em contrapartida, a presença de câncer e o uso de furosemida durante a hospitalização reduziram as chances de hipercorreção. Em conformidade com estudos observacionais recentes, a SDO foi rara e a mortalidade foi significativamente menor em pacientes que apresentaram hipercorreção. É possível que a hipercorreção da hiponatremia funcione simplesmente como um indicador de causas rapidamente reversíveis de hiponatremia, refletindo, em última análise, diagnósticos subjacentes menos propensos a provocar óbito. Portanto, são necessários ensaios clínicos randomizados antes que as salvaguardas atualmente estabelecidas pelas diretrizes para hiponatremia sejam abandonadas. Esses ensaios devem abordar algumas das limitações do nosso estudo e incluir uma avaliação formal dos sintomas e do estado volêmico para orientar a correção do sódio.

## Data Availability

Todos os dados gerados ou analisados durante este estudo estão incluídos neste artigo e nos seus arquivos de material suplementar. Quaisquer perguntas adicionais podem ser dirigidas ao autor correspondente. O conjunto de dados que sustenta os achados deste estudo não está disponível publicamente por conter informações que poderiam comprometer a privacidade dos participantes da pesquisa, mas está disponível junto ao PNR mediante solicitação razoável.

## Material Suplementar

O seguinte material online está disponível para este artigo:

Figura S1 - Distribuição de sódio sérico na admissão.

Figura S2 - Categorização da evolução do sódio sérico nas primeiras 24 horas.

Figura S3 - Sódio sérico na admissão, em 24 horas e em 48 horas estratificado por mortalidade hospitalar.

Tabela S1 - Frequência de hipercorreção do sódio sérico em 362 pacientes internados com hiponatremia grave.

Tabela S2 - Dados demográficos, clínicos e de desfecho de 362 pacientes internados com hiponatremia grave estratificados por mortalidade.
